# Myocardial Fibrosis in Cardiovascular Disease: An Integrative Biomarker–Imaging Framework Linking Molecular Mechanisms to Structural Phenotypes

**DOI:** 10.3390/jcm15103742

**Published:** 2026-05-13

**Authors:** Mateusz Świątko, Jakub Marek Baran, Aleksandra Czernicka, Łukasz Dudek, Maria Szewczyk, Jan Pietruszka, Łukasz Łazarowicz, Wacław Kochman, Ewelina A. Dziedzic

**Affiliations:** 1 Department of Cardiology, Bielański Hospital, 01-809 Warsaw, Poland; 2 Faculty of Medicine, Collegium Medicum, Cardinal Stefan Wyszyński University in Warsaw, 01-938 Warsaw, Poland; 3 Cardiovascular Clinic, Centre of Postgraduate Medical Education (CMKP), 01-813 Warsaw, Poland

**Keywords:** myocardial fibrosis, extracellular matrix remodeling, collagen turnover, procollagen type I C-terminal propeptide (PICP), procollagen type III N-terminal propeptide (PIIINP), collagen type I C-terminal telopeptide (CITP), matrix metalloproteinases (MMPs), galectin-3, osteopontin, soluble ST2 (sST2), N-terminal pro-B-type natriuretic peptide (NT-proBNP), periostin, microRNA, cardiac magnetic resonance (CMR), ventricular remodeling

## Abstract

**Background**: Myocardial fibrosis (MF) is a dynamic remodeling process characterized by excessive extracellular matrix (ECM) deposition, fibroblast activation, and dysregulated matrix turnover. Although initially reparative, persistent fibrotic remodeling promotes myocardial stiffening, electrical instability, and progressive cardiac dysfunction across diverse cardiovascular diseases. Circulating biomarkers reflecting collagen synthesis, degradation, proteolytic regulation, and inflammatory activation have emerged as potential tools for assessing fibrotic activity and risk stratification. **Methods**: This targeted narrative review was based on manually guided searches of PubMed and Scopus, supplemented by citation chaining and inclusion of landmark mechanistic and translational studies. Publications addressing myocardial extracellular matrix remodeling, circulating fibrosis-related biomarkers and imaging-derived fibrosis phenotypes were selected for qualitative synthesis. **Results**: Myocardial fibrosis reflects interconnected inflammatory, neurohormonal, oxidative, and extracellular matrix remodeling pathways. Among circulating biomarkers, C-terminal propeptide of procollagen type I (PICP) showed the most consistent association with myocardial collagen burden and adverse outcomes, whereas carboxy-terminal telopeptide of type I collagen (CITP), matrix metalloproteinases (MMPs), tissue inhibitors of metalloproteinases (TIMPs), galectin-3, osteopontin, soluble suppression of tumorigenicity 2 (sST2), and natriuretic peptides provided more context-dependent signals. Standalone interpretation remains limited by restricted cardiac specificity, renal dysfunction, systemic inflammation, assay heterogeneity, and lack of standardized thresholds. Integration with cardiac magnetic resonance (CMR)-derived late gadolinium enhancement (LGE), T1 mapping, and extracellular volume (ECV) may improve biological and structural phenotyping. **Conclusions**: Circulating biomarkers capture complementary dimensions of myocardial remodeling but cannot replace structural imaging. We propose an updated, hypothesis-generating biomarker–imaging framework integrating inflammatory activation, collagen turnover, matrix quality, hemodynamic stress, and structural imaging to support phenotypic stratification and future validation of antifibrotic strategies.

## 1. Introduction

Myocardial fibrosis (MF) represents a central and convergent mechanism leading to structural and functional deterioration of the heart across a broad spectrum of cardiovascular diseases. These include myocardial infarction, coronary heart disease, heart failure, cardiomyopathies, myocarditis, and arrhythmias [1,2,3,4,5,6,7,8]. Initially, fibrotic remodeling constitutes an adaptive reparative response to myocardial injury; however, when sustained, it becomes maladaptive, contributing to progressive ventricular stiffening, electrical instability, impaired oxygen diffusion, and adverse clinical outcomes [6,7]. Regardless of the initiating trigger, excessive extracellular matrix (ECM) deposition and altered matrix turnover ultimately form a structural substrate for heart failure progression and arrhythmogenesis [6,8,9].

At the mechanistic level, MF is a dynamic and self-perpetuating process driven by persistent inflammatory signaling, neurohormonal activation, and oxidative stress [10,11,12]. Injury-related stimuli promote fibroblast activation and differentiation into myofibroblasts via key profibrotic pathways, particularly transforming growth factor-β (TGF-β)-dependent signaling [10,11]. Activated myofibroblasts enhance synthesis of type I and III collagens, while dysregulation of matrix metalloproteinases (MMPs) and their tissue inhibitors of metalloproteinases (TIMPs) disrupts physiological matrix degradation [13,14,15]. Progressive ECM expansion, qualitative alterations in collagen cross-linking, and impaired matrix turnover increase myocardial stiffness, disturb mechano-transduction and electrical coupling, and reinforce inflammatory and profibrotic feedback loops [6,12].

Clinically, assessment of myocardial fibrosis remains challenging. Cardiac imaging modalities, particularly cardiac magnetic resonance (CMR), allow identification of focal replacement fibrosis through late gadolinium enhancement (LGE) and estimation of diffuse interstitial expansion using T1 mapping and extracellular volume (ECV) quantification [16,17,18]. However, imaging primarily reflects structural burden and established matrix accumulation. Conversely, circulating biomarkers—including markers of collagen synthesis, degradation and cross-linking, proteolytic regulation, inflammatory activation and hemodynamic stress—may capture biological remodeling activity that precedes or accompanies structural change [16,17,19,20].

**Figure 1:**
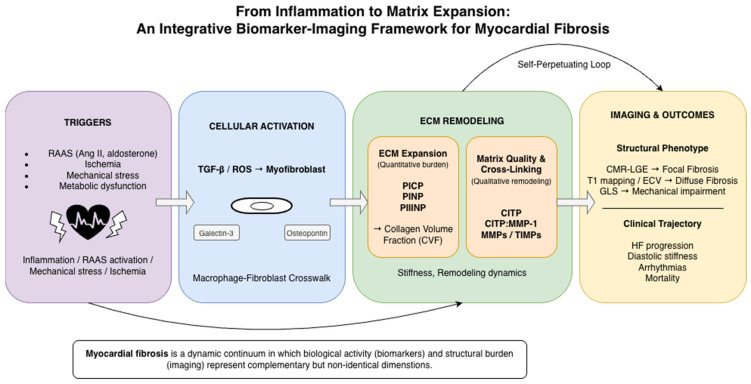
Integrative biomarker–imaging framework linking extracellular matrix remodeling with structural phenotypes and clinical outcomes. The schematic illustrates the continuum from triggers (e.g., RAAS activation, inflammation, mechanical stress) through cellular activation and extracellular matrix remodeling (collagen synthesis and matrix quality) to imaging-derived fibrosis phenotypes and clinical trajectories.

These modalities are often interpreted separately in spite of interrogating complementary dimensions of the same remodeling continuum. Building on previous work linking circulating fibrosis biomarkers with imaging-derived myocardial remodeling phenotypes, this review provides an updated and expanded biomarker–imaging framework that incorporates established, contextual, and emerging biomarkers across mechanistic, clinical, imaging, and therapeutic dimensions (Figure 1) [21,22,23].

## 2. Materials and Methods

This article was designed as a targeted narrative review integrating mechanistic, experimental, translational, and clinical evidence on myocardial extracellular matrix remodeling, circulating fibrosis-related biomarkers, and cardiac imaging-derived fibrosis phenotypes. This review should therefore be interpreted as a targeted narrative synthesis, not as a systematic review, meta-analysis, or formal evidence map.

Literature identification was performed through manually guided searches of PubMed and Scopus for publications available between January 2015 and February 2025. Earlier landmark studies were included when they provided essential mechanistic, pathophysiological, or clinical context. The review was not based on a single predefined Boolean search strategy applied uniformly across databases. Instead, iterative thematic searches were performed using combinations of MeSH terms and free-text keywords.

The core search vocabulary included: “myocardial fibrosis”, “cardiac fibrosis”, “extracellular matrix remodeling”, “extracellular matrix”, “collagen turnover”, “collagen synthesis”, “collagen degradation”, “procollagen”, “PICP”, “procollagen type I C-terminal propeptide”, “PINP”, “procollagen type I N-terminal propeptide”, “PIIINP”, “procollagen type III N-terminal propeptide”, “CITP”, “carboxy-terminal telopeptide of collagen type I”, “matrix metalloproteinases”, “MMP”, “MMP-1”, “MMP-9”, “tissue inhibitors of metalloproteinases”, “TIMP”, “TIMP-1”, “galectin-3”, “osteopontin”, “cardiac magnetic resonance”, “CMR”, “late gadolinium enhancement”, “LGE”, “T1 mapping”, “extracellular volume”, “ECV”, “heart failure”, “HFpEF”, “HFrEF”, “myocardial infarction”, “coronary heart disease”, “cardiomyopathy”, “myocarditis”, “atrial fibrillation”, and “arrhythmia”.

These terms were combined according to the thematic focus of each subsection, including fibrosis biology, collagen turnover biomarkers, inflammatory profibrotic mediators, and imaging-derived fibrosis phenotypes. The literature search was iterative and manually guided, without a formally structured systematic strategy.

Studies were selected based on relevance to myocardial fibrosis, inclusion of biomarker or imaging data, and translational or clinical applicability. Priority was given to studies with direct myocardial tissue validation, cardiac magnetic resonance-based fibrosis assessment, prospective clinical design, or biomarker–outcome associations. Case reports and studies without direct relevance to myocardial remodeling were generally not considered.

These terms were not applied as a reproducible Boolean search string across databases. Because this review was not based on a predefined, reproducible search strategy with formal inclusion and exclusion criteria, a PRISMA-style flow diagram was not constructed. The absence of a PRISMA flow diagram reflects the narrative and hypothesis-generating nature of this review rather than a systematic evidence synthesis approach.

No formal risk-of-bias assessment was performed. However, the evidentiary strength of individual sources was considered narratively. Greater interpretative weight was assigned to studies with direct myocardial tissue validation, CMR-based fibrosis assessment, prospective clinical design, biomarker–outcome associations, or interventional biomarker analyses. Preclinical studies and reviews were used primarily to support mechanistic interpretation, whereas clinical studies were used to evaluate translational relevance and limitations.

During revision, targeted supplementary searches were also performed for biomarker classes highlighted by the editor, including “soluble ST2”, “sST2”, “BNP”, “NT-proBNP”, “periostin”, “tenascin-C”, “fibulin-1”, “microRNA”, “miR-21”, and “miR-29”, combined with “myocardial fibrosis”, “cardiac fibrosis”, “extracellular matrix remodeling”, “heart failure”, and “cardiac magnetic resonance”. These searches were used to determine whether these markers should be incorporated into the conceptual framework or discussed as emerging/contextual biomarkers.

Therefore, the number of articles cited in the final synthesis should not be interpreted as the number of publications meeting predefined systematic eligibility criteria. Rather, the 138 cited references represent a purposively selected evidence base used to support a mechanistic and translational narrative synthesis. The final reference base included mechanistic, translational, observational clinical, interventional, and selected review literature relevant to the scope of the manuscript.

The main search domains, representative free-text terms, and revision-stage supplementary biomarker categories are summarized in Supplementary Table S1. A complete classification of all included references by study type, topic domain, and DOI is provided in Supplementary Table S2. The evidentiary composition of proportions of experimental, clinical observational, interventional, and review literature is summarised in Supplementary Table S3.

## 3. Results

### 3.1. Pathogenesis of Myocardial Fibrosis

MF is a reactive remodeling response to myocardial injury, involving fibroblast activation and ECM deposition that initially supports tissue repair [1,24]. In the healthy heart, cardiomyocytes are embedded within a structured extracellular matrix predominantly composed of collagen fibers synthesized by fibroblasts. Type I collagen accounts for approximately 80% of total collagen content [11]. Under physiological conditions, the cardiac ECM provides a structural scaffold ensuring mechanical stability and coordinated electrical conduction, while also serving as a reservoir for growth factors [25].

However, excessive ECM production and deposition lead to myocardial fibrosis [1]. This process is characterized by increased fibroblast activity, disproportionate accumulation of type I and III collagen, and impaired collagen degradation. Although initially adaptive, progressive remodeling ultimately becomes maladaptive and irreversible [14].

The transition from physiological to pathological fibrosis is well illustrated in myocardial infarction. Impaired tissue perfusion results in cardiomyocyte death and triggers an inflammatory response involving immune cell recruitment and fibroblast activation [10]. Necrotic cardiomyocytes release damage-associated molecular patterns (DAMPs), activating innate immune signaling and promoting cytokine-mediated fibroblast activation. Among these mediators, transforming growth factor-β (TGF-β) plays a central role in driving fibroblast-to-myofibroblast transition and extracellular matrix synthesis [10]. Activated myofibroblasts subsequently secrete large amounts of collagen and matrix proteins to compensate for tissue loss.

Neurohormonal activation, particularly through the renin–angiotensin–aldosterone system (RAAS), together with inflammatory cytokines, further amplifies fibroblast activation and collagen synthesis [10]. Pathological ECM accumulation results from both increased synthesis and reduced degradation. Dysregulation of MMPs and their TIMPs contributes to impaired matrix turnover [26].

Cardiac fibroblasts differentiate into myofibroblasts and exhibit increased ECM gene expression and altered metalloproteinase activity through SMAD-dependent TGF-β signaling, MAPK activation, and PI3K/Akt pathways [13]. Activated fibroblasts enhance procollagen synthesis, collagen maturation, and cross-linking, while simultaneously altering matrix degradation processes [14]. Hypertrophic cardiomyocytes further promote fibroblast proliferation and ECM production [27].

Canonical TGF-β signaling involves receptor-mediated SMAD activation and transcription of profibrotic genes, whereas non-canonical pathways include MAPK signaling and reactive oxygen species (ROS) generation [28]. MAPK pathways (including ERK1/2 and JNK1/2) and oxidative stress further enhance fibroblast proliferation, collagen maturation, and extracellular matrix accumulation while contributing to impaired matrix degradation through MMP/TIMP imbalance [11,12].

The consequence of these processes is architectural distortion and myocardial remodeling which, given the limited regenerative capacity of the heart, leads to progressive cardiac dysfunction [1,2,3,4]. Myocardial fibrosis should therefore be regarded not as a static structural abnormality but as a dynamic, self-perpetuating process. Persistent inflammatory and neurohormonal signaling promotes fibroblast activation and excessive matrix deposition, increasing myocardial stiffness, disrupting mechano-transduction and electrical coupling, and further amplifying profibrotic signaling. Pathological fibrosis can develop in myocardial infarction, chronic hypertension, aortic stenosis, arrhythmias, or primary cardiomyopathies [5]. Ultimately, cardiac fibrosis leads to myocardial stiffening, impaired oxygen diffusion, and progressive deterioration of cardiac function [6].

### 3.2. Clinical Context of Myocardial Fibrosis

Microscopic analysis of fibrotic tissue deposition patterns allows MF to be classified into two distinct types: reactive fibrosis and reparative (replacement) fibrosis. Reactive fibrosis is characterized by collagen accumulation within the interstitium and/or perivascular space, increasing diffusion distance and potentially contributing to cardiomyocyte hypoxia. Conversely, reparative fibrosis replaces areas of cardiomyocyte necrosis [29].

Reactive fibrosis typically results from excessive activation of resident fibroblasts and develops in the absence of significant cardiomyocyte loss [11,15,24]. Reparative replacement fibrosis occurs following cardiomyocyte loss and represents scar formation, in which collagen-rich tissue substitutes for irreversibly damaged myocardium [15,24]. Such remodeling may contribute to alterations in chamber geometry and ventricular structure in advanced disease states [11]. Across cardiovascular conditions, the relative contribution of reactive versus replacement fibrosis differs, creating distinct phenotypic patterns and specific “windows” for assessment using circulating biomarkers and cardiac magnetic resonance imaging [14,30].

#### 3.2.1. Myocardial Infarction

The myocardium exhibits limited regenerative capacity; therefore, sudden cardiomyocyte loss following myocardial infarction (MI) leads to a multifaceted reparative response and scar formation mediated by fibroblasts and myofibroblasts [31,32]. Excessive fibroblast activity can promote pathological fibrosis and adverse myocardial remodeling when mechanisms regulating fibroblast deactivation are disrupted [33]. Replacement fibrosis formed after MI constitutes a structural substrate that contributes to ventricular arrhythmias and progression toward heart failure [31,32,34].

Post-infarction healing proceeds through coordinated inflammatory, proliferative, and maturation phases, involving clearance of necrotic tissue, activation of innate immune signaling, fibroblast proliferation and differentiation into myofibroblasts, and ultimately collagen cross-linking and scar stabilization [32,33]. During the early inflammatory phase, fibroblasts interact with recruited immune cells, including macrophages, which regulate debris clearance and facilitate transition toward reparative, TGF-β-mediated fibrotic remodeling [32,35,36]. In the proliferative phase, fibroblasts migrate from non-infarcted regions and differentiate into myofibroblasts in response to mechanical stress, with distinct phenotypic profiles described during infarct healing [31,33].

Myofibroblasts are absent in healthy myocardium; however, their ability to migrate and contract—mediated in part by α-smooth muscle actin (α-SMA)—underlies their central role in scar formation and ECM deposition [31,32,33]. Increased deposition of type I and III collagen within the infarcted region contributes to impaired systolic and diastolic performance and adverse ventricular remodeling [15,31,32].

Neurohormonal activation, particularly the RAAS, together with TGF-β signaling, represents a central axis driving fibroblast-to-myofibroblast transition and profibrotic remodeling after MI [32,33,37]. The subsequent stage of scar maturation involves stabilization of the cross-linked collagen matrix and resolution of granulation tissue, leading to formation of a mechanically stable scar [32,33].

From a biomarker perspective, the early post-infarction phase is associated with increased circulating markers of collagen synthesis (e.g., PICP, PINP, and PIIINP), reflecting active scar formation. Subsequent scar maturation and matrix remodeling may be partially captured by changes in degradation-related markers such as CITP and indices reflecting collagen cross-linking, providing indirect insight into scar stability and long-term remodeling dynamics [17,19,38,39,40].

#### 3.2.2. Coronary Heart Disease

Coronary heart disease (CHD) is a chronic ischemic disorder resulting from progressive atherosclerotic narrowing of the coronary arteries and impaired myocardial perfusion [41]. Unlike myocardial infarction with abrupt cardiomyocyte loss leading to replacement scar formation, chronic CHD may promote diffuse interstitial remodeling through recurrent ischemia, endothelial dysfunction, microvascular rarefaction and low-grade inflammation [42].

Stable or chronic ischemic disease is often associated with myocardial fibrosis that is less sharply demarcated than post-infarction scar [42]. Diffuse interstitial fibrosis may develop in viable but chronically underperfused myocardium, especially in patients with comorbid hypertension, diabetes, renal dysfunction or heart failure [42,43,44,45]. This overlap complicates the interpretation of circulating fibrosis biomarkers in CHD, because elevations in collagen-turnover and profibrotic inflammatory markers may reflect combined effects of ischemia, ventricular remodeling, renal dysfunction, systemic inflammation, and concurrent cardiovascular disease rather than CHD-specific collagen deposition alone [20,46,47,48,49,50].

CHD-specific biomarker evidence remains limited, but available data support this cautious interpretation. Lepojärvi et al. evaluated serum PINP, PIIINP, galectin-3 (Gal-3), and soluble ST2 (sST2) in relation to LGE-CMR and echocardiographic diastolic filling parameters in patients with stable coronary artery disease (CAD), without prior myocardial infarction [51]. The study suggested association of Gal-3 with greater CMR-defined myocardial fibrosis, whereas Gal-3, soluble ST2 and PIIINP were higher in patients with impaired diastolic filling. However, PINP, PIIINP, and soluble ST2 did not show direct correlations with CMR-defined fibrosis strata, suggesting that circulating biomarkers in CHD may reflect mixed structural–functional remodeling rather than isolated collagen deposition.

From an imaging perspective, LGE is most useful for detecting local replacement scar related to prior infarction. T1 mapping and ECV quantification are better suited to assessing diffuse interstitial expansion in chronic ischemic remodeling [16,19]. Because biomarker data isolated to CHD without coexisting heart failure or prior infarction remain comparatively sparse [51], CHD is considered in this review mainly as a contributor to ischemic remodeling within broader heart failure and post-infarction fibrosis phenotypes.

#### 3.2.3. Heart Failure

Heart failure (HF) is a chronic syndrome resulting from impaired myocardial contractility and/or increased ventricular stiffness, leading to inadequate tissue perfusion and elevated filling pressures [52,53].

MF represents a central structural mechanism contributing to HF, encompassing both heart failure with preserved ejection fraction (HFpEF) and heart failure with reduced ejection fraction (HFrEF) [7]. In HF, fibrotic remodeling reflects the convergence of inflammatory and profibrotic signaling pathways activated across diverse cardiac stress conditions [54].

Collagen deposition is a key determinant of myocardial stiffness and dysfunction. Type I collagen forms thick, high-tensile fibers, whereas type III collagen forms thinner, more compliant fibers, and their relative proportion differs between HF phenotypes [30]. Endomyocardial biopsy data suggest that HFpEF is frequently associated with a relative predominance of type I collagen, while advanced HFrEF may demonstrate a relative increase in type III collagen [30]. Quantitatively, fibrosis is reflected by increased collagen volume fraction (CVF), which may vary according to regional wall stress and microvascular alterations [7].

Deposition of cross-linked collagen within the interstitium and vascular walls contributes to diastolic dysfunction and is considered a key mechanism in HFpEF, where increased myocardial stiffness and impaired relaxation often precede overt clinical deterioration [55]. By comparison, HFrEF is typically characterized by eccentric remodeling and chamber dilation secondary to cardiomyocyte loss, with disruption of the collagen scaffold further compromising systolic performance [56].

HFpEF is characterized by interstitial and perivascular fibrosis that contributes to adverse structural and functional phenotypes, with increasing fibrotic burden associated with impaired relaxation and ventricular stiffening [8]. Differences in collagen subtype composition, cross-linking patterns, and inflammatory drivers may partly explain the heterogeneous performance of circulating fibrosis biomarkers—such as PICP, PIIINP, CITP, and Gal-3—across HF populations.

Sex may act as an important biological modifier of myocardial fibrosis and heart failure phenotypes. HFpEF appears to be more prevalent in women, and sex-related differences in ventricular geometry, microvascular dysfunction, inflammatory activation, myocardial stiffness, collagen turnover, and biomarker profiles may influence the interpretation of both imaging-derived fibrosis measures and circulating fibrosis-related biomarkers [57,58,59]. These observations suggest that future biomarker–imaging studies should include sex-stratified analyses and evaluate whether fibrosis biomarkers, imaging thresholds, or integrated models require sex-specific interpretation.

Integration of biomarker profiles with imaging-derived measures of fibrosis, particularly CMR-based ECV, may improve phenotypic characterization and risk stratification.

#### 3.2.4. Myocarditis

Myocarditis is an inflammatory disease of the myocardium which may progress to fibrotic remodeling and ventricular dysfunction [60]. Myocardial inflammation and perivascular fibrosis impair oxygen and nutrient delivery, thereby perpetuating pathological remodeling [4]. Inflammatory cell infiltration may evolve toward a profibrotic phenotype, linking acute myocardial injury to subsequent structural remodeling [60,61].

Experimental models of autoimmune myocarditis have demonstrated that TGF-β signaling mediates the transition from inflammation to fibrosis [62]. Rho-associated coiled-coil-containing protein kinases (ROCKs) further amplify TGF-β-dependent profibrotic signaling, thereby exacerbating myocardial fibrosis [63]. The ST2 protein has also been implicated in fibrotic progression; its soluble form (sST2), produced by stressed cardiac fibroblasts, may enhance profibrotic signaling and oxidative stress, accelerating matrix remodeling [64].

Clinically, the inflammatory-to-fibrotic transition in myocarditis may be partially reflected by elevated circulating markers of fibroblast activation and collagen turnover, including Gal-3 and sST2 [64,65]. However, their specificity for myocarditis-related fibrosis remains limited. Integration of circulating biomarkers with CMR-based tissue characterization may improve differentiation between active inflammation and established fibrotic remodeling [29,66,67].

#### 3.2.5. Cardiomyopathies

Dilated cardiomyopathy (DCM) is characterized by aberrant myocardial fibrosis, involving excessive collagen deposition and impaired matrix degradation [16]. Replacement fibrosis can be detected in a substantial proportion of patients with DCM using gadolinium-enhanced CMR, most commonly within the interventricular septum [68]. Fibrotic remodeling in DCM is driven by mechanical stress, microvascular ischemia, and activation of immune and neurohormonal pathways, with increased profibrotic mediators such as angiotensin II and aldosterone [68].

Fibrosis is also a key histological feature of hypertrophic cardiomyopathy (HCM). In HCM, replacement fibrosis is typically heterogeneous and asymmetric, frequently involving the interventricular septum and anterior free wall. Greater fibrotic burden has consistently been associated with adverse clinical outcomes, irrespective of anatomical distribution [69]. In addition, myocardial fibrosis in HCM has been strongly linked to increased arrhythmic risk [70].

Patients with diabetes and hypertension commonly exhibit concentric remodeling, increased ECV, and enhanced interstitial fibrosis [43]. Diabetic cardiomyopathy is defined as myocardial dysfunction occurring in the absence of overt coronary artery disease, hypertension, or valvular pathology and is characterized by excessive extracellular matrix deposition [44]. Diabetic cardiomyopathy may present with distinct phenotypes and is linked to TGF-β activation, hyperglycemia-driven fibroblast stimulation, endothelial-to-mesenchymal transition, accumulation of advanced glycation end-products, and alterations in MMP/TIMP balance, collectively promoting matrix expansion and collagen cross-linking [43,44,45].

In infiltrative cardiomyopathies, chronic inflammation promotes progressive fibrosis and myocardial dysfunction [71]. Similarly, arrhythmogenic cardiomyopathy (ACM) is characterized by replacement of ventricular myocardium with fibrous or fibro-fatty tissue, predisposing to ventricular arrhythmias and sudden cardiac death [72].

Across cardiomyopathies, fibrosis burden assessed by CMR-derived late gadolinium enhancement and extracellular volume correlates with clinical outcomes [68,69]. However, integration of imaging-derived fibrosis measures with circulating biomarkers of collagen turnover remains an area of ongoing translational investigation, with potential to refine risk stratification and phenotypic characterization [66,67].

#### 3.2.6. Arrhythmias

MF plays a central role in arrhythmogenesis. Accumulation of electrically inactive collagen and expansion of fibroblast populations disrupt electrical wave propagation and facilitate reentry, creating a proarrhythmic substrate [9,73,74]. Both diffuse interstitial and patchy replacement fibrosis contribute to increased susceptibility to ventricular tachyarrhythmias.

In mitral valve prolapse (MVP), inferobasal fibrosis has been identified as a structural substrate associated with ventricular arrhythmias and mechanical dysfunction [75]. Replacement fibrosis is generally considered more arrhythmogenic than reactive interstitial fibrosis, as it more profoundly disrupts myocardial conduction pathways and structural continuity [76].

Fibrosis-related arrhythmias are further influenced by myofibroblast–cardiomyocyte electrical coupling. Through electrotonic interactions and modulation of conduction properties, myofibroblasts can enhance arrhythmic vulnerability [27,34]. Mechanical stress and recurrent arrhythmia episodes may further stimulate fibrotic remodeling, creating a self-perpetuating cycle of fibrosis and electrical instability [27].

Multiple experimental and clinical studies support a strong association between fibrosis and atrial fibrillation (AF). Atrial fibrosis is increasingly recognized as a key determinant of AF recurrence, treatment resistance, and complication risk [76]. Atrial fibrosis has been particularly well characterized using delayed-enhancement cardiac magnetic resonance imaging (DE-CMR). In the DECAAF multicenter prospective study, the extent of left atrial fibrosis quantified by DE-CMR was independently associated with atrial arrhythmia recurrence after catheter ablation, supporting atrial fibrotic burden as an imaging-defined marker of arrhythmogenic substrate [77]. However, DECAAF II showed that targeting MRI-defined fibrotic areas in addition to pulmonary vein isolation did not significantly reduce atrial arrhythmia recurrence compared with pulmonary vein isolation alone and was associated with a higher rate of safety events [78]. Therefore, atrial DE-CMR is best interpreted as a tool for substrate characterization and risk stratification rather than as a validated stand-alone guide for fibrosis-targeted ablation. Arrhythmogenic cardiomyopathy (ACM) involves progressive replacement of myocardium with fibrous or fibro-fatty tissue, creating an unstable electrical substrate predisposing to malignant ventricular arrhythmias and sudden cardiac death [28,79].

Circulating markers of collagen turnover and fibroblast activation have been explored as indirect indicators of arrhythmogenic substrate, particularly in atrial fibrillation; however, their ability to discriminate electrical from purely structural remodeling remains limited and requires integration with imaging-based fibrosis assessment. Markers reflecting collagen cross-linking and matrix turnover—such as indices derived from CITP and MMP-1—may provide additional insight into qualitative changes in extracellular matrix architecture that influence conduction properties, although their role in arrhythmic risk stratification remains to be fully established [19,39,40].

### 3.3. Circulating Biomarkers of Myocardial Fibrosis: A Translational Perspective

#### 3.3.1. Biomarkers of Collagen Synthesis (PICP, PINP, PIIINP)

Expansion of the ECM is a hallmark of myocardial fibrotic remodeling driven by increased synthesis and maturation of fibrillar collagens. Circulating biomarkers released during procollagen processing provide an indirect readout of collagen production and may complement imaging-based assessment of diffuse fibrosis, particularly when interpreted alongside CMR-derived ECV fraction and histology-derived CVF.

Although PICP, PINP, and PIIINP are frequently grouped as procollagen-derived biomarkers of collagen formation, they should not be interpreted as interchangeable markers of the same biological pathway. PICP and PINP are related to type I collagen formation, whereas PIIINP reflects type III procollagen processing and may capture a partly distinct remodeling signal. These biomarkers differ in collagen subtype specificity, release kinetics, tissue sources, assay characteristics, and susceptibility to renal and extracardiac confounding. Therefore, their interpretation should account for collagen subtype biology and clinical context rather than assuming equivalent diagnostic or prognostic meaning [17,20,38,47,80] (Table 1).

##### C-Terminal Propeptide of Procollagen Type I (PICP)

PICP (also referred to as PIP) is released during the extracellular conversion of type I procollagen into mature type I collagen, reflecting collagen type I synthesis [17,81]. Clinical and experimental data indicate that serum PICP correlates with myocardial collagen burden, including CVF and histologically assessed type I collagen volume fraction in patients with heart failure and hypertension. Cardiac release of PICP into the circulation has been demonstrated, reinforcing its relative specificity for cardiac ECM remodeling rather than solely systemic collagen turnover [17]. Accordingly, PICP has been proposed as a clinically relevant marker of myocardial fibrosis in heart failure [17]. In hypertrophic cardiomyopathy, plasma PICP quantitatively reflects myocardial fibrosis, and correlations between myocardial and circulating PICP concentrations further support its translational relevance [18].

The HOMAGE study linked PICP with structural and functional cardiac phenotypes. Among patients receiving spironolactone, higher PICP was associated with left ventricular hypertrophy, left atrial enlargement, and increased ventricular stiffness, whereas reductions in PICP paralleled decreases in E/e′, consistent with improved diastolic function. Such associations were not observed for PIIINP or Gal-3 in the same analysis [82]. In diabetic populations across different HF stages, both PICP and the PICP/PIIINP ratio were higher in HFmrEF compared with HFpEF, suggesting that PICP may capture phenotypic differences in ECM remodeling severity and progression [85].

Analyses from EMPEROR-Preserved and EMPEROR-Reduced further underscore the prognostic dimension of collagen synthesis markers. Higher PICP concentrations were associated with older age, more advanced HF, atrial fibrillation, and chronic kidney disease. Importantly, PICP showed the strongest association with clinical endpoints, including HF hospitalization or cardiovascular death [20]. Collectively, PICP demonstrates the most consistent association with myocardial collagen burden, structural remodeling, and adverse HF outcomes, supporting its potential role in risk stratification and monitoring of antifibrotic response.

##### N-Terminal Propeptide of Procollagen Type I (PINP)

PINP represents the aminoterminal extension of type I procollagen and is cleaved in equimolar amounts alongside PICP during fibril formation; it circulates predominantly in its trimeric form [80]. As a marker of type I collagen synthesis, PINP has been associated with remodeling-related phenotypes. Among patients with metabolic syndrome and atrial fibrillation, elevated PINP levels were linked to more severe atrial fibrosis and correlated with other fibrotic markers, including Gal-3 [86]. In studies evaluating eplerenone-related reduction in atrial fibrillation risk, decreases in PINP and PIIINP accompanied improvements in electrocardiographic indices associated with atrial fibrosis, supporting its potential role in dynamic atrial remodeling assessment [87].

However, cardiac specificity is limited. In addition, delayed release kinetics and assay-related variability may attenuate cross-sectional signal, leading to inconsistent findings despite potential utility in longitudinal or interventional settings [38].

##### N-Terminal Propeptide of Procollagen Type III (PIIINP)

PIIINP is generated during processing of fibrillar procollagen and enters the circulation via the lymphatic system. Owing to its stability and favorable storage properties, it has been widely studied in HF populations. Higher circulating concentrations have been associated with adverse hemodynamic profiles, poor prognosis, and increased risk of advanced HF outcomes, including transplantation and mortality [38]. In resistant hypertension treated with spironolactone (ASCOT subanalysis), PIIINP was the collagen synthesis marker most closely aligned with the study endpoint, suggesting responsiveness to antifibrotic-oriented interventions [83].

However, cardiac specificity is limited and elevations may reflect extracardiac remodeling. Methodological issues, including incomplete separation of PIIINP from intact procollagen, may lead to underestimation of type III collagen synthesis [38].

##### Translational Integration with Imaging and Clinical Endpoints

Biomarkers of collagen synthesis provide an indirect window into ECM expansion, yet their translational utility depends on cardiac specificity, remodeling stage, and comorbidity burden. Among available markers, PICP shows the most consistent association with myocardial collagen burden and adverse outcomes [17,20,82], whereas PINP and PIIINP appear more context-dependent [20,38,47,86,87]. Their integration with imaging-derived measures of diffuse fibrosis is addressed in Section 3.3.5.

#### 3.3.2. Biomarkers of Collagen Degradation and Cross-Linking (CITP, CITP:MMP-1)

While biomarkers of collagen synthesis reflect ECM expansion, markers of collagen degradation and cross-linking provide complementary insight into matrix turnover and structural remodeling dynamics. Among these, the C-terminal telopeptide of type I collagen (CITP) and the CITP:MMP-1 ratio have emerged as candidates for assessing not only collagen breakdown but also the qualitative properties of collagen fibers, including the degree of cross-linking, which directly influences myocardial stiffness.

##### Collagen Type I C-Terminal Telopeptide (CITP)

CITP is released during degradation of mature type I collagen fibers and reflects the rate of collagen breakdown within the extracellular matrix [39]. In the context of myocardial fibrosis, circulating CITP levels may reflect ongoing remodeling activity rather than static collagen. However, cardiac specificity is limited.

This distinction is particularly relevant in chronic heart failure, where persistent turnover of collagen fibers contributes to progressive architectural distortion and altered ventricular compliance.

Evidence suggests that alterations in collagen degradation are linked to atrial and ventricular remodeling. In patients with persistent AF undergoing electrical cardioversion, higher baseline CITP levels were observed in individuals who experienced AF recurrence compared with those maintaining sinus rhythm. These findings imply that increased collagen turnover, as captured by CITP, may indicate structural remodeling predisposing to arrhythmia persistence [88]. Similarly, in patients with chronic heart failure and reduced ejection fraction treated with β-blockers, improvements in left ventricular ejection fraction were associated with reductions in circulating CITP levels, suggesting that reverse remodeling can be paralleled by decreased collagen degradation activity [89].

However, CITP alone does not fully capture the mechanical consequences of fibrosis. The degree of collagen cross-linking plays a crucial role in determining myocardial stiffness, particularly in HFpEF, where increased cross-linked type I collagen contributes to diastolic dysfunction.

##### CITP:MMP-1 Ratio and Collagen Cross-Linking

The ratio of CITP to matrix metalloproteinase-1 (MMP-1) has been proposed as an indirect index of collagen cross-linking. Since MMP-1 cleaves type I collagen at specific sites, a relative reduction in CITP generation despite MMP-1 presence may indicate increased cross-linking and resistance of collagen fibers to enzymatic degradation [19,40]. In hypertensive heart failure, excessive myocardial collagen cross-linking was associated with increased risk of hospitalization for heart failure, whereas the CITP:MMP-1 ratio showed an inverse correlation with adverse outcomes, supporting its potential role as a marker of qualitative collagen remodeling rather than simply quantitative accumulation [19].

In the HOMAGE study, higher CITP:MMP-1 ratios were associated with lower degrees of collagen cross-linking and more pronounced reverse remodeling following spironolactone therapy, suggesting that dynamic modulation of cross-linking may be therapeutically relevant [40]. Importantly, collagen cross-linking is often incompletely captured by standard imaging parameters such as ECV or native T1 mapping, which primarily reflect matrix expansion rather than fiber quality; therefore, the CITP:MMP-1 ratio may complement imaging by identifying mechanical phenotypes characterized by increased stiffness and impaired diastolic function despite modest ECV elevation.

##### Matrix Turnover, Stiffness, and Remodeling Dynamics

From a pathophysiological perspective, increased collagen cross-linking reduces myocardial compliance and contributes to elevated filling pressures and diastolic stiffness. This distinction is particularly relevant in HFpEF phenotypes, where biomarkers reflecting degradation resistance (low CITP relative to MMP-1) may provide insight into matrix stiffness. Conversely, elevated CITP levels in settings of active remodeling may reflect ongoing matrix disruption and maladaptive turnover rather than irreversible collagen accumulation.

However, cardiac specificity is limited. Therefore, CITP and related indices should be interpreted in conjunction with structural and functional assessments, including echocardiographic parameters of diastolic function and CMR-derived measures of diffuse fibrosis.

##### Clinical Endpoints and Translational Implications

CITP and the CITP:MMP-1 ratio provide information on collagen degradation, cross-linking, and matrix stiffness rather than collagen synthesis alone. These markers may be particularly relevant in phenotypes characterized by altered myocardial compliance and diastolic dysfunction. Their relationship to imaging-defined fibrosis, functional remodeling, and phenotype mapping is discussed in Section 3.3.5.

#### 3.3.3. Regulatory Mediators of ECM Remodeling (MMPs, TIMPs)

Dynamic remodeling of the myocardial ECM depends not only on collagen synthesis and degradation but also on tightly regulated proteolytic control. MMPs and their endogenous inhibitors, TIMPs, constitute the principal regulatory axis governing ECM turnover. Rather than acting as simple degradative enzymes, these mediators modulate stage-specific remodeling responses and influence long-term structural adaptation.

##### Matrix Metalloproteinases (MMPs): Proteolysis and Remodeling Activity

MMPs are zinc-dependent endoproteases responsible for degradation of extracellular matrix proteins, including fibrillar collagens [90]. Their role in cardiac remodeling is context-dependent, as specific MMP subtypes may exert pro- or anti-fibrotic effects depending on remodeling stage and inflammatory milieu [91]. Individual MMPs cleave defined collagen subtypes, thereby shaping matrix architecture and turnover dynamics; for example, MMP-1 degrades type I collagen, while MMP-9 has been implicated in inflammation-associated remodeling (Table 2).

Experimental data suggest that MMP-12 deficiency attenuates angiotensin II-induced cardiac fibrosis and reduces profibrotic signaling, highlighting the interaction between proteolysis and inflammation [126]. However, the principal translational relevance of MMPs lies in their association with structural and functional phenotypes in human disease.

In hypertrophic cardiomyopathy, elevated MMP-9 concentrations have been associated with late gadolinium enhancement on cardiac magnetic resonance imaging and with adverse cardiovascular outcomes, suggesting parallelism between proteolytic activity and imaging-defined fibrosis burden [92]. In heart failure, circulating MMP levels correlate with restrictive left ventricular filling patterns, supporting an association between proteolytic remodeling and diastolic dysfunction [93]. In dilated cardiomyopathy, higher MMP-1 levels have been linked to poorer clinical outcomes, and the CITP:MMP-1 ratio correlates with global longitudinal strain, integrating biochemical indices with functional measures of myocardial performance [16].

These findings indicate that circulating MMPs may reflect remodeling dynamics rather than static collagen accumulation. In a preclinical model of sunitinib-induced cardiac fibrosis, sacubitril/valsartan attenuated fibrotic signaling and oxidative stress, suggesting potential antifibrotic mechanisms that require clinical validation [127].

However, cardiac specificity is limited. Accordingly, MMPs should be interpreted within a broader clinical and imaging framework.

##### Tissue Inhibitors of Metalloproteinases (TIMPs): Balance and Matrix Stabilization

TIMPs form high-affinity 1:1 complexes with MMPs, thereby maintaining ECM integrity and limiting excessive proteolysis [95,96]. Four major subtypes (TIMP-1 to TIMP-4) regulate MMP activity, with TIMP-3 exhibiting broad inhibitory capacity, including effects on ADAM and ADAMTS proteases [91,97]. Beyond protease inhibition, TIMPs modulate fibroblast phenotype and inflammatory signaling, underscoring their regulatory role in remodeling biology [99].

Altered TIMP expression has been documented across cardiovascular phenotypes. Increased TIMP-1 levels have been associated with pathological remodeling in arterial hypertension and hypertensive heart failure [48,94], and in HFpEF, elevated TIMP-1 correlates with diastolic dysfunction and disturbed collagen metabolism [48]. Conversely, reduced TIMP-1 expression in rheumatic valvular tissue has been linked to increased TGF-β1 expression and collagen volume fraction, illustrating context-dependent dysregulation of proteolytic balance [98]. Experimental models further demonstrate stage-dependent variation in MMP-1, MMP-9, and TIMP-1 expression across remodeling phases [128], supporting the concept of temporally regulated proteolytic control.

##### Translational Perspective and Clinical Integration

The MMP/TIMP axis represents a regulatory layer of ECM remodeling that complements synthesis markers and degradation/cross-linking indices. However, cardiac specificity is limited. The integration of MMPs and TIMPs with imaging-derived fibrosis parameters and remodeling phenotypes is discussed in Section 3.3.5.

#### 3.3.4. Inflammation-Driven Profibrotic Mediators (Galectin-3, Osteopontin)

Beyond structural markers of collagen turnover, a distinct class of circulating biomarkers reflects inflammatory-driven fibroblast activation. Among these, Gal-3 and osteopontin represent key mediators linking immune activation, macrophage–fibroblast crosstalk, endothelial-to-mesenchymal transition (EndMT), and neurohormonal signaling to myocardial fibrotic remodeling.

##### Galectin-3: Macrophage–Fibroblast Crosstalk and RAAS Amplification

Gal-3, a β-galactoside-binding lectin, is currently regarded as a central mediator of cardiac inflammation and fibrosis [65,103]. Although it exhibits intracellular functions related to transcriptional regulation and cell survival, its extracellular role is most relevant in myocardial remodeling. Extracellular Gal-3 modulates cell–cell and cell–matrix interactions and facilitates growth factor signaling by stabilizing receptor complexes, including those involving TGF-β and VEGF [46,102].

From a mechanistic perspective, Gal-3 acts at the interface between immune activation and fibroblast response. Activated macrophages release Gal-3, which promotes fibroblast proliferation and differentiation into collagen-producing myofibroblasts, thereby driving interstitial fibrosis [102]. In myocardial infarction, Gal-3 contributes to macrophage infiltration and polarization, amplifying inflammatory signaling and subsequent profibrotic pathways [101,129]. Experimental models have consistently demonstrated increased Gal-3 expression during pressure overload (TAC models), angiotensin II stimulation, and post-infarction remodeling, with attenuation of fibrosis observed after pharmacological or genetic inhibition of Gal-3 [129,130,131,132,133].

Importantly, Gal-3 also interacts with the RAAS. Aldosterone overexpression enhances Gal-3-mediated cardiac and vascular fibrosis, and suppression of upstream profibrotic signaling (e.g., via Src inhibition) reduces Gal-3 expression and downstream collagen accumulation [100,132], supporting its role as an amplifier of neurohormonal profibrotic cascades.

##### Clinical Correlations and Imaging Integration

Clinically, elevated circulating Gal-3 levels have been reported in heart failure populations and correlate with adverse remodeling and outcome risk [46,103]. A Gal-3 assay has received FDA 510(k) clearance for use, together with clinical evaluation, as an aid in assessing prognosis in patients with chronic heart failure [101,105,106]. In HF cohorts, Gal-3 has demonstrated predictive value for long-term outcomes, particularly in patients with preserved ejection fraction [46,104].

However, the relationship between Gal-3 and direct measures of myocardial collagen burden is complex. While Gal-3 correlates with certain remodeling phenotypes and clinical outcomes, its association with histologically assessed collagen volume fraction or circulating collagen synthesis markers is inconsistent [49]. Some studies suggest that Gal-3 may better reflect inflammatory-driven remodeling and microvascular dysfunction rather than pure collagen deposition [49]. Imaging-based analyses have shown that Gal-3 may be associated with focal remodeling patterns detected by magnetic resonance imaging [66], yet its performance as a direct surrogate of diffuse fibrosis remains limited.

However, its interpretation is constrained by limited cardiac specificity.

Collectively, Gal-3 appears to reflect inflammatory-driven profibrotic activation rather than quantitative collagen burden alone. Its translational value may lie in identifying patients with active inflammatory remodeling who are at increased risk of adverse outcomes.

##### Osteopontin: EndMT and Macrophage-Mediated Fibrotic Signaling

Osteopontin is a matricellular protein increasingly recognized as a mediator of fibrotic remodeling in cardiovascular disease [107]. It is secreted by activated macrophages, particularly M2-polarized subsets, and promotes fibroblast-to-myofibroblast differentiation, angiogenesis, and ECM remodeling [35,108].

At the cellular level, osteopontin contributes to macrophage–fibroblast crosstalk. Transcriptomic analyses indicate that SPP1-expressing macrophages coordinate fibroblast activation in heart failure and chronic kidney disease, with osteopontin interacting with CD44 and integrin pathways to promote extracellular matrix expansion [109,110].

Additionally, osteopontin has been implicated in EndMT, a process contributing to fibroblast accumulation in fibrotic myocardium [111].

Experimental studies demonstrate that osteopontin deficiency impairs reparative fibrosis after myocardial infarction, leading to excessive ventricular dilation and reduced collagen deposition [35]. Conversely, pathological overexpression of osteopontin is associated with oxidative stress, TGF-β activation, and diastolic dysfunction [107]. These dual observations highlight that osteopontin participates in both adaptive scar formation and maladaptive chronic fibrosis, depending on context and timing.

##### Translational Considerations and Limitations

Gal-3 and osteopontin capture upstream profibrotic signaling rather than structural ECM burden per se. Their integration with imaging-derived fibrosis parameters and remodeling phenotypes is discussed in Section 3.3.5, while limitations related to specificity, renal function, and systemic inflammation are addressed in Section 3.3.6.

##### Additional Emerging and Contextual Biomarkers

Several additional biomarkers warrant consideration, although they should not be interpreted as equivalent to direct collagen-turnover markers. sST2, a soluble isoform of the IL-33/ST2 signaling axis, reflects myocardial stress, inflammation, and adverse remodeling. It has established prognostic relevance in heart failure but does not directly quantify collagen deposition [114,115].

BNP and NT-proBNP are also not fibrosis biomarkers in the strict molecular sense. They primarily reflect myocardial wall stress, filling pressures, and hemodynamic burden. Their inclusion remains clinically relevant because ventricular stiffness, structural remodeling, and extracellular matrix expansion often coexist with elevated natriuretic peptide concentrations. BNP and NT-proBNP primarily reflect hemodynamic load and contextualize the functional consequences of remodeling rather than fibrosis itself [116,118].

Matricellular proteins, including periostin, tenascin-C, and fibulin-1, participate in extracellular matrix organization, fibroblast activation, inflammation, and tissue repair. Among these, periostin has the strongest fibrosis-oriented rationale because it is re-expressed in injured myocardium, produced by activated cardiac fibroblasts/myofibroblasts, and associated with myocardial fibrosis in failing human hearts and with adverse post-myocardial infarction remodeling [119,122]. Tenascin-C is also re-expressed during myocardial injury and inflammation, whereas fibulin-1 has been linked to extracellular matrix remodeling, restrictive filling patterns, and pressure-overload phenotypes [121,122,134]. Despite their biological relevance, clinical translation remains limited by smaller cardiovascular cohorts, assay heterogeneity, incomplete standardization, and less consistent linkage to routine imaging-derived fibrosis phenotypes than established collagen-turnover biomarkers.

Non-coding RNAs, particularly miR-21 and miR-29, are mechanistically relevant to fibroblast activation and extracellular matrix gene regulation. However, miRNA-based fibrosis assessment remains investigational because of pre-analytical variability, platform heterogeneity, tissue specificity, and lack of validated clinical thresholds [123,124]. These biomarkers are therefore best regarded as emerging translational candidates rather than clinically established fibrosis tools.

#### 3.3.5. Integration with Cardiac Imaging

Circulating biomarkers of myocardial fibrosis provide indirect insight into ECM turnover; however, their clinical interpretation gains translational value when integrated with cardiac imaging. Advanced imaging modalities—particularly CMR with LGE, T1 mapping, ECV quantification, and echocardiographic strain analysis—allow structural and functional characterization of myocardial remodeling that complements circulating markers of collagen synthesis, degradation, and inflammatory activation.

##### Late Gadolinium Enhancement Versus Diffuse Fibrosis

LGE-CMR enables detection of focal replacement fibrosis and scar formation. In hypertrophic cardiomyopathy, circulating MMP-9 levels have been shown to correlate with LGE-defined fibrosis and adverse cardiovascular events, suggesting that proteolytic remodeling activity may parallel imaging-detected scar burden [92]. Similarly, associations between collagen turnover markers and functional imaging parameters such as global longitudinal strain have been reported in dilated cardiomyopathy, linking biochemical remodeling indices with structural and mechanical alterations [16].

However, LGE primarily captures focal fibrosis and may underestimate diffuse interstitial remodeling. Biomarkers of collagen synthesis (e.g., PICP) and degradation (e.g., CITP) may therefore reflect more global ECM dynamics.

##### T1 Mapping and Extracellular Volume

Quantification of diffuse fibrosis using T1 mapping and calculation of ECV offer a noninvasive estimate of interstitial matrix expansion. Although direct correlations between individual biomarkers and ECV are variably reported, synthesis markers such as PICP have demonstrated strong associations with histologically assessed CVF and myocardial type I collagen content [17,18], supporting their conceptual alignment with diffuse fibrosis assessment.

Conversely, certain inflammatory mediators such as Gal-3 show inconsistent correlations with histological collagen burden and circulating collagen synthesis markers [49]. Imaging studies suggest that Gal-3 may better reflect non-infarct-related remodeling patterns rather than direct quantitative collagen accumulation [66]. These findings indicate that imaging and biomarkers capture overlapping but non-identical dimensions of fibrotic remodeling—structural expansion versus biological activation.

##### Speckle Tracking and Functional Remodeling

Functional consequences of fibrosis, particularly impaired myocardial deformation and diastolic stiffness, can be assessed using echocardiographic parameters such as global longitudinal strain and restrictive filling patterns. In dilated cardiomyopathy, the CITP:MMP-1 ratio correlated with global longitudinal strain [16], suggesting that collagen cross-linking and degradation resistance influence myocardial mechanics. Similarly, circulating MMP levels have been associated with restrictive left ventricular filling patterns in heart failure [93], linking proteolytic remodeling to diastolic dysfunction.

These observations support the concept that biochemical markers of matrix turnover may reflect dynamic mechanical alterations before or alongside detectable structural changes on imaging.

##### Complementary Roles and Phenotype Mapping

Despite these associations, neither circulating biomarkers nor imaging alone provides a complete representation of myocardial fibrosis. Conversely, imaging techniques detect structural fibrosis but cannot fully characterize ongoing biological activity or early remodeling before measurable matrix expansion occurs.

Thus, circulating biomarkers and imaging modalities should be viewed as complementary tools capturing distinct dimensions of myocardial remodeling. Synthesis markers (e.g., PICP) may reflect ECM expansion aligned with diffuse fibrosis metrics such as ECV, degradation and cross-linking indices (e.g., CITP, CITP:MMP-1) may capture matrix quality and mechanical consequences, and inflammatory mediators (e.g., Gal-3, osteopontin) may indicate upstream profibrotic activation that precedes structural deposition.

Conceptually, synthesis biomarkers (e.g., PICP) align most closely with diffuse interstitial fibrosis quantified by native T1 mapping, ECV, and histological CVF; degradation and cross-linking indices (e.g., CITP, CITP:MMP-1) correspond to matrix quality and mechanical phenotypes such as increased stiffness and diastolic dysfunction; inflammatory mediators (e.g., Gal-3, osteopontin) reflect biologically active remodeling states that may precede measurable structural expansion; and LGE primarily captures focal replacement fibrosis and established scar.

This phenotype-oriented mapping underscores that biomarker classes and imaging modalities interrogate complementary dimensions of remodeling—matrix quantity, matrix quality, inflammatory activation, and irreversible scar—rather than redundant aspects of the same process.

From a translational perspective, integration of biochemical and imaging parameters may enable multidimensional characterization of myocardial fibrosis—distinguishing active remodeling from established scar, diffuse interstitial expansion from focal replacement fibrosis, and inflammatory activation from structural stiffness—thereby improving phenotyping and risk stratification in cardiovascular disease.

**Figure 2:**
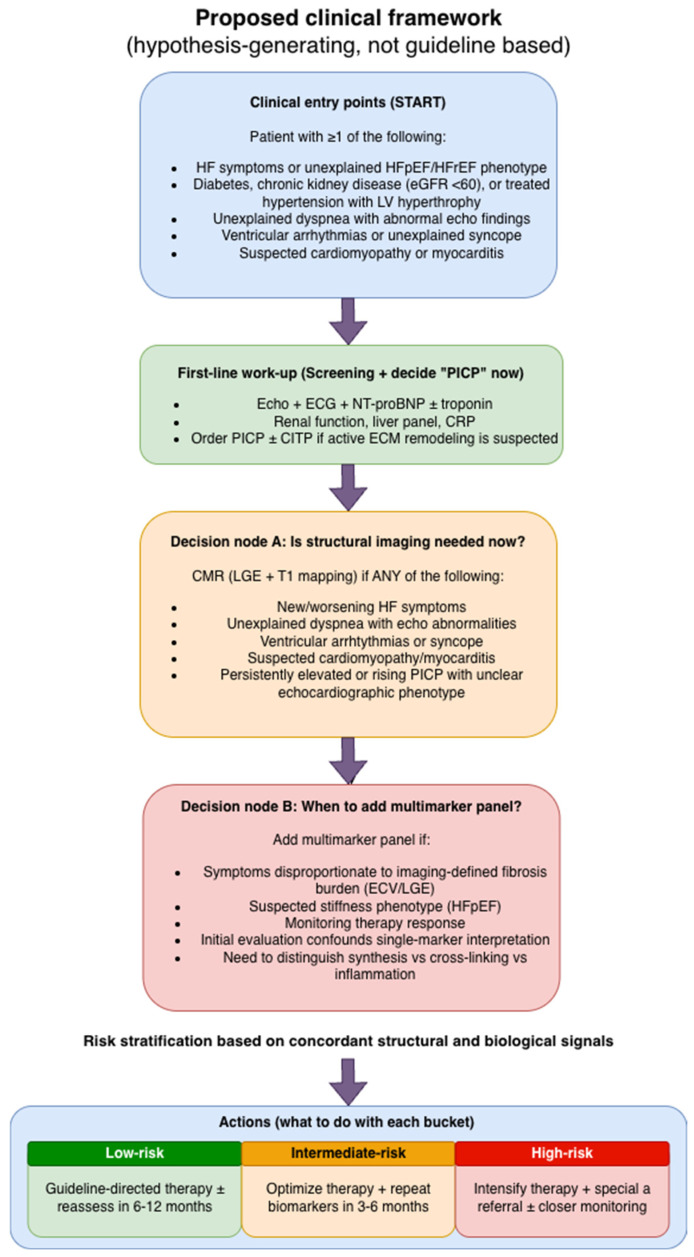
Proposed clinical biomarker–imaging workflow for myocardial fibrosis assessment and risk stratification. The flowchart outlines clinical entry points, first-line diagnostic evaluation, indications for advanced imaging (CMR), use of single biomarkers and multimarker panels, and risk-based management strategies.

Based on these considerations, a hypothesis-generating clinical workflow integrating biomarkers and imaging is proposed to support practical decision-making in patients with suspected myocardial fibrosis (Figure 2).

A particularly promising direction for future research involves prospective biomarker–CMR sub-studies embedded within randomized heart failure or cardiomyopathy trials. Such designs would enable direct correlation of dynamic biomarker changes with imaging-derived fibrosis phenotypes and clinical outcomes, facilitating calibration of biomarker thresholds, validation of multimarker panels, and development of clinically actionable biomarker–imaging algorithms.

In parallel, emerging antifibrotic therapeutic strategies—including pirfenidone, anti–TGF-β-targeted interventions, and sodium–glucose cotransporter 2 (SGLT2) inhibitors—provide a translational framework for testing the proposed model. Integration of biomarker and imaging endpoints in such trials may allow assessment of treatment response and identification of fibrosis-modifying effects beyond conventional clinical outcomes.

Finally, machine learning-based approaches to multimarker panel optimization represent a promising avenue for future investigation. Data-driven integration of biochemical, imaging, and clinical variables may enable identification of latent remodeling phenotypes and improve risk stratification beyond single-marker strategies, although such approaches require validation in large, well-characterized prospective cohorts.

To make the proposed framework more operational without implying clinical validation, biomarker classes can be mapped to dominant remodeling phases and corresponding imaging or functional correlates, as summarized in Table 3.

At present, no validated cut-off values, biomarker thresholds, composite scores, or biomarker–imaging decision rules exist for routine clinical use. The proposed framework should therefore be interpreted as hypothesis-generating and requires validation in prospective studies integrating standardized biomarker assessment with CMR-derived fibrosis parameters and predefined clinical endpoints. Evidence supporting this conceptual mapping is discussed in Section 3.3.1, Section 3.3.2, Section 3.3.3, Section 3.3.4 and Section 3.3.5. The table summarizes biomarker–imaging relationships reported across mechanistic, observational, imaging-correlative, and post hoc clinical studies. It is intended as a hypothesis-generating conceptual framework for organizing biomarker classes, remodeling phases, and imaging correlates, not as a validated diagnostic or therapeutic algorithm. No biomarker thresholds, cut-off values, composite scores, or prospectively validated decision rules are currently available.

Although this framework provides a structured integration of biomarker classes and imaging-derived phenotypes, it remains conceptual and hypothesis-generating. It cannot yet support routine clinical implementation or biomarker–imaging-based decision-making. The proposed mapping should therefore be interpreted as a conceptual and translational framework for organizing current evidence and guiding hypothesis-driven research, rather than as a validated diagnostic or therapeutic strategy.

Future validation will require prospective studies integrating multimarker panels with standardized CMR-based fibrosis assessment and predefined clinical endpoints. In particular, biomarker–imaging sub-studies embedded within heart failure or cardiomyopathy trials may enable calibration of biomarker thresholds, assessment of incremental prognostic value, and development of clinically actionable multimodal models.

#### 3.3.6. Clinical Applicability and Limitations

The limitations summarized here apply across biomarker classes and are therefore discussed collectively rather than repeated in detail within each individual biomarker subsection.

Although circulating biomarkers of myocardial fibrosis offer valuable insight into ECM remodeling, their clinical applicability remains limited by several important factors, including restricted organ specificity, influence of renal function and systemic inflammation, methodological variability, and lack of standardization.

The proposed biomarker–imaging framework remains conceptual and hypothesis-generating. No validated thresholds, cut-off values, composite scores, or biomarker–imaging decision rules can currently be recommended. The framework has not been prospectively tested against predefined clinical endpoints or externally validated in independent datasets. Therefore, it should be used to organize current evidence and guide future research. It should not be interpreted for routine diagnostic classification, therapeutic selection, or treatment monitoring.

##### Limited Organ Specificity

A major limitation of most fibrosis-related biomarkers is their lack of cardiac specificity. Collagen synthesis markers such as PIIINP and PINP may reflect systemic collagen turnover rather than myocardial fibrosis alone. For example, PIIINP has been shown not to consistently correlate with myocardial collagen mRNA expression and may instead reflect extracardiac tissue injury in heart failure [47]. Similarly, elevated PIIINP levels have been reported in pulmonary arterial hypertension, where fibrotic and vascular remodeling extend beyond the myocardium [84].

Gal-3 also illustrates this limitation. Although involved in cardiac inflammation and remodeling, circulating Gal-3 levels do not consistently correlate with histological myocardial collagen content or circulating collagen synthesis markers in hypertension-related heart failure [49]. These findings suggest that Gal-3 may reflect broader inflammatory and microvascular processes rather than direct quantitative collagen accumulation.

MMPs and TIMPs are likewise expressed in multiple tissues and participate in systemic remodeling and inflammatory responses. Therefore, circulating concentrations may represent global proteolytic activity rather than cardiac-specific ECM turnover [94,135]. As a consequence, interpretation of these markers requires careful phenotypic and imaging context.

##### Influence of Renal Function

Renal function significantly influences circulating concentrations of several fibrosis biomarkers. Elevated Gal-3 levels have been associated with reduced estimated glomerular filtration rate (eGFR) and microalbuminuria in heart failure populations [46]. Moreover, in EMPEROR analyses, higher PICP and PINP concentrations were associated with chronic kidney disease [20], indicating that comorbid renal dysfunction may confound interpretation of collagen synthesis markers.

These observations are particularly relevant in heart failure populations, where renal impairment is common and may independently alter biomarker kinetics and clearance.

Clinically, renal dysfunction should be considered a core contextual variable in the interpretation of fibrosis-related biomarkers. Future studies should report eGFR alongside fibrosis biomarker concentrations and explicitly account for renal function when interpreting renal-sensitive biomarkers such as Gal-3, PICP, PINP, MMPs, and osteopontin [20,21,94,108,115]. These approaches should be prospectively validated before routine clinical implementation.

In addition to these considerations, several pragmatic strategies may help mitigate the impact of renal dysfunction in clinical and research settings. These include adjustment for eGFR, use of biomarker ratios less dependent on renal clearance (e.g., CITP:MMP-1), and development of renal-adjusted reference ranges for selected biomarkers. Such approaches may improve interpretability of fibrosis-related biomarkers in multimorbid populations, although they require prospective validation before routine clinical application.

##### Influence of Systemic Inflammation and Comorbidities

Inflammation represents another confounding factor. Gal-3 and osteopontin are secreted by activated macrophages and are involved in systemic inflammatory pathways [35,109]. Elevated osteopontin levels have been observed in various inflammatory and infectious contexts, including HIV- and SIV-associated models of fibrosis [50], underscoring its role beyond cardiac-specific remodeling.

Similarly, MMPs are regulated by inflammatory stimuli and may be modulated by age, sex, and environmental factors [10]. Consequently, circulating levels may reflect generalized inflammatory activation rather than isolated myocardial fibrosis.

##### Lack of Methodological Standardization

Differences in assay methodology and biomarker kinetics further limit comparability across studies. For example, cross-sectional studies using radioimmunoassay have reported limited changes in PINP concentrations despite evidence of increased myocardial fibrosis, potentially due to delayed release kinetics [38]. Furthermore, incomplete separation of PIIINP from procollagen may lead to underestimation of type III collagen synthesis and misinterpretation of results [38].

Variability in assay platforms (e.g., ELISA versus radioimmunoassay) and differences in cut-off values across studies reduce the ability to define universally applicable thresholds for clinical decision-making.

##### Potential of Multimarker Panels

Given these limitations, a single biomarker is unlikely to capture the multidimensional nature of myocardial fibrosis. The available evidence suggests that different biomarkers reflect distinct components of remodeling:

- PICP appears to most consistently correlate with myocardial collagen burden and adverse outcomes [17,20,82].
- CITP and the CITP:MMP-1 ratio provide insight into collagen degradation and cross-linking dynamics [16,19].
- MMPs and TIMPs reflect regulatory control of proteolysis and remodeling stage.
- Gal-3 and osteopontin capture inflammatory-driven profibrotic activation.

Integration of these biomarkers into multimarker panels, interpreted alongside imaging-derived measures such as CMR-based fibrosis assessment and functional parameters of diastolic stiffness, may improve phenotypic stratification and risk prediction. Rather than serving as standalone diagnostic tools, circulating markers may therefore function as complementary indicators of biological activity within a broader, multimodal evaluation framework.

Importantly, the proposed biomarker–imaging framework should be interpreted as a conceptual and hypothesis-generating translational model rather than a prescriptive clinical algorithm. It is based predominantly on mechanistic insights and observational associations rather than prospective randomized validation. Therefore, it does not represent guideline-endorsed recommendations and should not substitute individualized clinical decision-making. Prospective studies are required to determine whether integrated biomarker–imaging strategies improve patient outcomes.

## 4. Conclusions and Future Perspectives

Myocardial fibrosis is not merely a structural scar but a biologically active and self-amplifying remodeling process driven by persistent inflammation, fibroblast activation, and dysregulated ECM turnover. The resulting expansion and qualitative alteration of the matrix form the structural substrate for ventricular stiffening, electrical instability, and progressive heart failure across diverse cardiovascular phenotypes.

Circulating biomarkers capture distinct layers of this remodeling continuum. Collagen synthesis markers (e.g., PICP) reflect ECM expansion; degradation and cross-linking indices (e.g., CITP and the CITP:MMP-1 ratio) provide insight into matrix quality and stiffness; regulatory mediators (MMPs, TIMPs) indicate proteolytic control; and inflammatory factors such as Gal-3 and osteopontin signal upstream profibrotic activation. However, no single biomarker sufficiently represents the complexity of myocardial fibrosis. Among circulating biomarkers, PICP currently provides the most consistent reflection of myocardial collagen burden, whereas other markers should be interpreted as complementary indicators of distinct remodeling processes.

Cardiac imaging remains indispensable for defining structural burden. Late gadolinium enhancement identifies focal replacement fibrosis, whereas T1 mapping and extracellular volume quantify diffuse interstitial remodeling. Functional parameters such as myocardial strain further translate matrix alterations into mechanical consequences. Biomarkers and imaging should therefore be viewed as complementary tools—biochemical signals reflect biological activity, while imaging defines anatomical and functional expression.

The major limitation of currently available biomarkers lies in their limited cardiac specificity and susceptibility to renal dysfunction, systemic inflammation, and extracardiac collagen turnover. Methodological heterogeneity and absence of standardized thresholds further restrict routine implementation.

**Figure 3:**
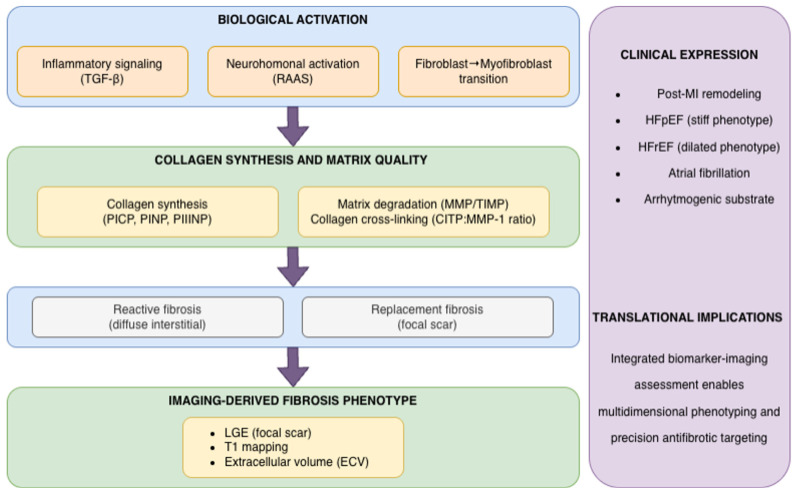
Translational continuum from biological activation to imaging-derived fibrosis phenotypes. The diagram summarizes the progression from inflammatory and neurohormonal activation through collagen synthesis and matrix remodeling to reactive and replacement fibrosis, as reflected by cardiac imaging modalities such as LGE and extracellular volume quantification.

The proposed translational framework linking biological activation, collagen turnover, and imaging-defined fibrosis phenotypes is summarized in Figure 3.

Future validation of this framework should proceed through prospective, multimodal study designs. A feasible approach would be a multicenter cohort enrolling patients across fibrosis-prone phenotypes, including HFpEF, HFrEF, post-infarction remodeling, hypertrophic cardiomyopathy, dilated cardiomyopathy, and atrial fibrillation. Participants should undergo standardized biomarker profiling together with CMR-based LGE, native T1 mapping, extracellular volume quantification, and echocardiographic strain assessment. Prespecified endpoints should include fibrosis progression, heart failure hospitalization, arrhythmia recurrence, cardiovascular mortality, and treatment response. A complementary strategy would be a prospective biomarker sub-study embedded within a CMR-based imaging trial to test whether biomarker changes track changes in ECV, LGE burden, myocardial stiffness, and clinical outcomes.

Therapeutically, the framework may support phenotypic enrichment, monitoring of biological response, and surrogate endpoint development in antifibrotic or remodeling-modifying trials. Relevant contexts include pirfenidone in CMR-defined HFpEF fibrosis, SGLT2 inhibitors with reported effects on collagen-turnover markers in EMPEROR analyses, mineralocorticoid receptor antagonism, neprilysin inhibition, and emerging anti-TGF-β-directed strategies [20,22,136,137]. Integrated biomarker–imaging profiles could help identify patients with active collagen turnover, inflammatory profibrotic activation, or established structural fibrosis, while linking biological remodeling activity with measurable structural change. However, biomarker-guided therapeutic decisions require prospective validation before clinical implementation.

Finally, artificial intelligence and machine learning may help optimize multimodal phenotyping by identifying nonlinear relationships among circulating biomarkers, imaging-derived fibrosis phenotypes, renal function, sex, comorbidities, and outcomes. AI-based CMR analysis may also support automated segmentation and quantitative assessment of structural remodeling, although such models require transparent reporting and external validation before clinical implementation [138].

## Figures and Tables

**Table 1:** Distinction between type I and type III procollagen-derived biomarkers.

Biomarker	Collagen Subtype	Distinctive Biological and Interpretative Features
PICP	Type I collagen	C-terminal propeptide; shows the most consistent myocardial fibrosis signal among these markers, but still reflects collagen formation beyond the heart [17,20,81,82].
PINP	Type I collagen	N-terminal propeptide; related to the same collagen subtype as PICP, but differs in circulating forms, kinetics, assay behavior, and extracardiac contribution [20,38,80].
PIIINP	Type III collagen	N-terminal propeptide; may capture broader tissue, vascular, inflammatory, or extracardiac remodeling signals and should not be interpreted as a direct analogue of PICP [38,47,83,84].

PICP, PINP, and PIIINP are grouped here as procollagen-derived biomarkers, but they differ in collagen subtype specificity, release kinetics, tissue sources, renal handling, and assay behavior. They should therefore be interpreted as related but non-interchangeable markers of collagen formation and remodeling. Evidence supporting this distinction is discussed in Section 3.3.1 [17,20,38,47,80].

**Table 2:** Circulating Biomarkers of Myocardial Fibrosis: Mechanistic, Structural and Clinical Integration.

Biomarker	Pathophysiological Domain	Molecular Mechanism	Correlation with Myocardial Structure (Histology/CMR)	Association with Functional Impairment	Prognostic Value	Major Limitations	Level of Clinical Validation
PICP	Collagen synthesis	Released during type I procollagen cleavage; reflects type I collagen formation and ECM expansion [17,81]	Strongly associated with myocardial CVF and histological collagen content; associated with structural remodeling [17,18]	Associated with LV hypertrophy, increased stiffness, and diastolic dysfunction [82,85]	Predicts HF hospitalization and cardiovascular mortality; therapy-responsive [20,82]	Systemic collagen turnover; renal function; inter-assay variability; lack of standardized thresholds [17,20]	Observational; biomarker sub-analyses of RCT cohorts (HOMAGE, EMPEROR)
PINP	Collagen synthesis	N-terminal propeptide of type I procollagen; equimolar release with PICP [38,80]	Associated with atrial fibrosis and remodeling [86,87]	Associated with AF burden and advanced HF [20,86]	Associated with disease severity; less consistent prognostic value than PICP [20]	Extracardiac type I collagen turnover, renal clearance (CKD); delayed kinetics; inter-assay heterogeneity [20,38,80]	Observational; post hoc analyses
PIIINP	Collagen synthesis (type III)	Marker of type III collagen synthesis; released via lymphatic circulation [38]	Variably associated with myocardial fibrosis; inconsistent association with collagen mRNA expression [47]	Associated with hemodynamic impairment and HF severity [38,83]	Associated with adverse outcomes in HF and PAH [83,84]	Systemic type III collagen turnover, renal dysfunction and systemic inflammation; assay heterogeneity [38,47]	Observational studies
CITP	Collagen degradation	Released during type I collagen breakdown; reflects matrix turnover [39]	Associated with remodeling parameters and strain indices [16,67]	Associated with diastolic dysfunction and LV functional changes [88,89]	Higher levels associated with AF recurrence and adverse remodeling [19,88,89]	Systemic collagen degradation; renal clearance; assay variability [19,47]	Observational studies
CITP:MMP-1 ratio	Collagen cross-linking	Inverse marker of collagen cross-linking and matrix stiffness [19,40]	Associated with global longitudinal strain and remodeling [16,40]	Associated with LV stiffness and remodeling dynamics [19,40]	Predicts HF hospitalization and cardiovascular mortality [19]	Requires combined measurement; lack of standardized assays and thresholds; limited prospective validation; potential renal influence [19,40]	Observational; mechanistic-clinical translational studies
MMP-1/MMP-9	ECM degradation control	Zinc-dependent proteases regulating collagen cleavage and ECM remodeling [90,91]	Associated with fibrosis extent in CMR and biopsy-based studies [16,92]	Associated with restrictive filling pattern and adverse remodeling [93]	Higher levels associated with worse outcomes in DCM and HCM [16,92,94]	inflammation-sensitive vascular remodeling; stage-dependent expression; significant inter-assay variability [90,91]	Observational; experimental–clinical translational studies
TIMP-1	Proteolysis inhibition	Inhibits MMP activity; regulates ECM accumulation [95,96,97]	Associated with CVF and fibrotic remodeling [48,98]	Associated with diastolic dysfunction and hypertensive remodeling [48,94]	Elevated in progressive remodeling states [94,99]	inflammation and vascular remodeling; assay heterogeneity; potential renal influence [94,99]	Observational studies
Galectin-3	Inflammation-driven fibrosis	Macrophage–fibroblast crosstalk; amplifies TGF-β signaling and myofibroblast activation [46,65,100,101,102,103]	Associated with focal and diffuse remodeling; partial CMR correlation [66]	Associated with HF progression and remodeling [46,104]	FDA-cleared prognostic assay in chronic HF; predicts HFpEF development [101,104,105,106]	renal function, systemic inflammation; indirect relationship to collagen burden inter-assay variability [46,49,105]	Observational; regulatory-approved risk biomarker (HF)
Osteopontin	Profibrotic inflammatory mediator	Promotes fibroblast activation, macrophage recruitment, and EndMT [35,107,108,109,110]	Associated with interstitial and perivascular fibrosis [107,111]	Associated with adverse remodeling and diastolic dysfunction [107,112]	Associated with disease severity in HF and vascular pathology [50]	expressed in systemic inflammatory and vascular conditions; influenced by renal dysfunction; limited assay standardization [50,107]	Experimental and observational studies
sST2	Inflammatory–mechanical stress signaling	Soluble form of the IL-33 receptor; reflects myocardial stress, inflammation, and profibrotic signaling [113,114]	Indirectly associated with remodeling; indirect collagen burden marker [113,114]	Associated with HF severity and adverse remodeling phenotypes [114]	Prognostic biomarker in chronic HF; useful for risk stratification [114,115]	Not fibrosis-specific; does not quantify collagen deposition; influenced by systemic inflammation; no fibrosis-specific thresholds [113,114,115]	observational and post hoc HF analyses
BNP/NT-proBNP	Hemodynamic stress	Released in response to myocardial stretch and elevated wall stress [116,117]	Indirectly associated with structural remodeling and chamber stress [118]	Strongly associated with filling pressures, HF severity, and functional limitation [116,117]	Established diagnostic and prognostic markers in HF [116,117]	Not fibrosis-specific; influenced by age, renal function, rhythm, obesity, and loading conditions [116]	Guideline-established diagnostic and prognostic HF biomarkers
Periostin/tenascin-C/fibulin-1	Matricellular remodeling proteins	ECM organization, fibroblast activation, tissue repair, and inflammatory remodeling; periostin has the strongest fibrosis-oriented rationale within this group [119,120]	Mechanistically linked to matrix remodeling; periostin has direct human failing-heart association with myocardial fibrosis, while clinical imaging correlations for the group remain less standardized [119,120,121,122]	Potentially associated with remodeling severity [121,122]	Emerging prognostic relevance, but less validated [121]	Limited assay standardization; smaller clinical datasets; limited routine availability [119,120,121,122]	Experimental and early translational studies
miR-21 and miR-29	Post-transcriptional regulation of fibrosis	miR-21 promotes profibrotic signaling; miR-29 regulates ECM gene expression [123,124]	Experimental and translational association with fibrotic remodeling [123,124]	Potential association with remodeling phenotypes [125]	Investigational [125]	Pre-analytical variability; platform heterogeneity; no validated clinical thresholds [125]	Experimental and early translational studies

AF, atrial fibrillation; BNP, B-type natriuretic peptide; CITP, collagen type I C-terminal telopeptide; CKD, chronic kidney disease; CMR, cardiac magnetic resonance; CVF, collagen volume fraction; DCM, dilated cardiomyopathy; ECM, extracellular matrix; EMPEROR, Empagliflozin Outcome Trial in Patients with Chronic Heart Failure; EndMT, endothelial-to-mesenchymal transition; FDA, U.S. Food and Drug Administration; HCM, hypertrophic cardiomyopathy; HF, heart failure; HFpEF, heart failure with preserved ejection fraction; HOMAGE, Heart Omics in AGEing; IL-33, interleukin-33; LV, left ventricular; miR, microRNA; MMP, matrix metalloproteinase; MMP-1, matrix metalloproteinase-1; MMP-9, matrix metalloproteinase-9; mRNA, messenger ribonucleic acid; NT-proBNP, N-terminal pro-B-type natriuretic peptide; PAH, pulmonary arterial hypertension; PICP, procollagen type I C-terminal propeptide; PINP, procollagen type I N-terminal propeptide; PIIINP, procollagen type III N-terminal propeptide; RCT, randomized controlled trial; sST2, soluble suppression of tumorigenicity 2; TGF-β, transforming growth factor beta; TIMP, tissue inhibitor of metalloproteinases; TIMP-1, tissue inhibitor of metalloproteinases-1. The level of clinical validation reflects the predominant type of evidence supporting each biomarker category in this narrative synthesis and should not be interpreted as a formal grading of evidence or as a validated clinical recommendation.

**Table 3:** Translational biomarker–imaging mapping across myocardial remodeling stages.

Remodeling Phase	Dominant Biological Process	Representative Biomarkers	Imaging/Functional Correlate	Suggested Clinical Interpretation/Use Case
Early profibrotic activation	Inflammation, fibroblast activation, neurohormonal signaling	Gal-3, sST2, miR-21/miR-29	Native T1, T2 mapping (inflammatory context), early ECV changes	Identification of biologically active remodeling before structural fibrosis; may support early risk stratification
Collagen synthesis/ECM expansion	Increased type I and III collagen production	PICP, PINP, PIIINP	Native T1, ECV, CVF (histology)	Suggests diffuse interstitial fibrosis progression; potential marker of disease activity and therapeutic response
Matrix degradation and turnover	Active collagen breakdown and ECM remodeling	CITP, MMP-1, MMP-9	ECV, strain, remodeling indices	Reflects dynamic remodeling rather than static fibrosis; may indicate ongoing structural adaptation
Cross-linking and stiffness	Collagen maturation, reduced degradability, increased stiffness	CITP:MMP-1 ratio	Diastolic function (E/e′), strain, stiffness indices	Identifies stiffness-dominant phenotypes (e.g., HFpEF); may refine functional characterization
Proteolytic regulation	Balance between ECM degradation and inhibition	MMPs, TIMPs	ECV, LGE, strain	Reflects regulatory balance of remodeling; may indicate stage-dependent ECM control
Hemodynamic consequence	Wall stress and chamber remodeling	BNP, NT-proBNP	LV/LA volumes, filling pressures, strain	Provides functional context of fibrosis-related remodeling; not fibrosis-specific
Established replacement fibrosis	Scar formation and irreversible remodeling	No specific circulating biomarker	LGE-CMR	Identifies advanced structural fibrosis and arrhythmogenic substrate

AF, atrial fibrillation; BNP, B-type natriuretic peptide; CITP, collagen type I C-terminal telopeptide; CMR, cardiac magnetic resonance; CVF, collagen volume fraction; ECM, extracellular matrix; ECV, extracellular volume; Gal-3, galectin-3; HFpEF, heart failure with preserved ejection fraction; LA, left atrial; LGE, late gadolinium enhancement; LV, left ventricular; miR, microRNA; MMP, matrix metalloproteinase; MMP-1, matrix metalloproteinase-1; MMP-9, matrix metalloproteinase-9; NT-proBNP, N-terminal pro-B-type natriuretic peptide; PICP, procollagen type I C-terminal propeptide; PINP, procollagen type I N-terminal propeptide; PIIINP, procollagen type III N-terminal propeptide; sST2, soluble suppression of tumorigenicity 2; TIMP, tissue inhibitor of metalloproteinases. This table presents a hypothesis-generating conceptual mapping of biomarker classes, remodeling phases, and imaging or functional correlates. It should not be interpreted as a validated diagnostic or therapeutic algorithm.

## Data Availability

No new data were created or analyzed in this study.

## Supplementary Materials

The following supporting information can be downloaded at: https://www.mdpi.com/article/10.3390/jcm15103742/s1, Table S1: Search vocabulary used in the targeted narrative review; Table S2: Reference classification by study type and topic domain (n = 138); Table S3: Reference classification by study type and topic domain (n = 138).

## Abbreviations

The following abbreviations are used in this manuscript:

ACM	Arrhythmogenic cardiomyopathy
ADAM	A Disintegrin and Metalloproteinase
ADAMTS	Disintegrin and Metalloproteinase with Thrombospondin Motifs
AF	Atrial fibrillation
AI	Artificial intelligence
α-SMA	Alpha-smooth muscle actin
ASCOT	Anglo-Scandinavian Cardiac Outcomes Trial
BNP	B-type natriuretic peptide
CAD	Coronary artery disease
CD44	Cluster of differentiation 44
CHD	Coronary heart disease
CITP	Carboxy-terminal telopeptide of type I collagen
CKD	Chronic kidney disease
CMR	Cardiac magnetic resonance
CVF	Collagen volume fraction
DAMPs	Damage-associated molecular patterns
DCM	Dilated cardiomyopathy
DE-CMR	Delayed-enhancement cardiac magnetic resonance
DECAAF	Delayed-Enhancement MRI Determinant of Successful Radiofrequency Catheter Ablation of Atrial Fibrillation
ECM	Extracellular matrix
ECV	Extracellular volume
E/e′	Ratio of early mitral inflow velocity to early diastolic mitral annular velocity
eGFR	Estimated glomerular filtration rate
ELISA	Enzyme-linked immunosorbent assay
EMPEROR	Empagliflozin Outcome Trial in Patients with Chronic Heart Failure
EndMT	Endothelial-to-mesenchymal transition
ERK1/2	Extracellular signal-regulated kinase 1/2
FDA	Food and Drug Administration
Gal-3	Galectin-3
HCM	Hypertrophic cardiomyopathy
HF	Heart failure
HFmrEF	Heart failure with mildly reduced ejection fraction
HFpEF	Heart failure with preserved ejection fraction
HFrEF	Heart failure with reduced ejection fraction
HIV	Human immunodeficiency virus
HOMAGE	Heart Omics in AGEing
IL-33	Interleukin-33
JNK1/2	c-Jun N-terminal kinase 1/2
LA	Left atrial
LGE	Late gadolinium enhancement
LV	Left ventricular
MAPK	Mitogen-activated protein kinase
MF	Myocardial fibrosis
MI	Myocardial infarction
MMP	Matrix metalloproteinase
MMP-1	Matrix metalloproteinase-1
MMP-9	Matrix metalloproteinase-9
MMP-12	Matrix metalloproteinase-12
MVP	Mitral valve prolapse
NT-proBNP	N-terminal pro-B-type natriuretic peptide
PAH	Pulmonary arterial hypertension
MeSH	Medical Subject Headings
PICP	C-terminal propeptide of procollagen type I
PI3K/Akt	Phosphoinositide 3-kinase/Protein kinase B signaling pathway
PINP	N-terminal propeptide of procollagen type I
PIIINP	N-terminal propeptide of procollagen type III
PIP	Procollagen type I C-terminal propeptide
RAAS	Renin–angiotensin–aldosterone system
RCT	Randomized controlled trial
ROCKs	Rho-associated coiled-coil-containing protein kinases
ROS	Reactive oxygen species
SGLT2	Sodium–glucose cotransporter 2
SIV	Simian immunodeficiency virus
SMAD	Intracellular signaling proteins mediating TGF-β signaling
sST2	Soluble suppression of tumorigenicity 2
SPP1	Secreted phosphoprotein 1
TAC	Transverse aortic constriction
TGF-β	Transforming growth factor beta
TIMP	Tissue inhibitor of metalloproteinases
TIMP-1	Tissue inhibitor of metalloproteinases 1
TIMP-3	Tissue inhibitor of metalloproteinases 3
TIMP-4	Tissue inhibitor of metalloproteinases 4
VEGF	Vascular endothelial growth factor
