# Supplementary material for: Myocardial Fibrosis in Cardiovascular Disease: An Integrative Biomarker–Imaging Framework Linking Molecular Mechanisms to Structural Phenotypes
Source: J Clin Med. 2026 May 13;15(10):3742. doi: 10.3390/jcm15103742 (PMC13207939; doi:10.3390/jcm15103742)
Supplement: Supplementary file 1 [file jcm-15-03742-s001.zip › jcm-4263386-supplementary.pdf]

Supplementary Table S1. Search vocabulary used in the targeted narrative review

Search domain	Representative terms
Fibrosis biology	myocardial fibrosis; cardiac fibrosis; extracellular matrix remodeling; collagen turnover; collagen synthesis; collagen degradation
Collagen biomarkers	PICP; procollagen type I C-terminal propeptide; PINP; procollagen type I N-terminal propeptide; PIIINP; procollagen type III N-terminal propeptide; CITP; carboxy-terminal telopeptide of collagen type I
Proteolytic regulation	matrix metalloproteinases; MMP; MMP-1; MMP-9; tissue inhibitors of metalloproteinases; TIMP; TIMP-1
Inflammatory/profibrotic mediators	galectin-3; osteopontin; soluble ST2; sST2
Imaging	cardiac magnetic resonance; CMR; late gadolinium enhancement; LGE; T1 mapping; extracellular volume; ECV
Clinical contexts	heart failure; HFpEF; HFrEF; myocardial infarction; coronary heart disease; cardiomyopathy; myocarditis; atrial fibrillation; arrhythmia
Revision-stage supplementary biomarkers	BNP; NT-proBNP; periostin; tenascin-C; fibulin-1; microRNA; miR-21; miR-29

Terms were combined iteratively according to the thematic focus of each manuscript section. They were not applied as a single reproducible Boolean search string across databases. This table summarizes the main search vocabulary used to support the targeted narrative synthesis and should not be interpreted as a systematic search strategy; BNP, B-type natriuretic peptide; CITP, carboxy-terminal telopeptide of collagen type I; CMR, cardiac magnetic resonance; ECV, extracellular volume; HFpEF, heart failure with preserved ejection fraction; HFrEF, heart failure with reduced ejection fraction; LGE, late gadolinium enhancement; MMP, matrix metalloproteinase; NT-proBNP, N-terminal pro-B-type natriuretic peptide; PICP, procollagen type I C-terminal propeptide; PINP, procollagen type I N-terminal propeptide; PIIINP, procollagen type III N-terminal propeptide; sST2, soluble suppression of tumorigenicity 2; TIMP, tissue inhibitor of metalloproteinase.

Supplementary Table S2. Reference classification by study type and topic domain (n = 138).

Reference number	First author	Year	DOI	Study Type	Topic or Domain
1.	Li et al.	2021	10.1016/j.lfs.2021.119186	Narrative / mechanistic review	Epigenetics-based therapeutics for myocardial fibrosis
2.	Xu et al.	2022	10.18632/aging.204070	Experimental – animal model	PTX3 depletion inhibits myocardial fibrosis in post-MI HF
3.	Huang & Dai	2023	10.1016/j.gene.2023.147705	Experimental – animal model	ATF3, autophagy and myocardial fibrosis remodeling after MI
4.	Quijada et al.	2023	10.1172/JCI162188	Experimental – animal / translational	Cardiac pericytes mediate remodeling response to MI
5.	McKinsey et al.	2023	10.1093/cvr/cvac142	Narrative / mechanistic review	Emerging epigenetic therapies of cardiac fibrosis and remodeling in HF
6.	Piek et al.	2016	10.1007/s10741-016-9536-9	Narrative / mechanistic review	The fibrosis–cell death axis in heart failure
7.	González et al.	2018	10.1016/j.jacc.2018.02.021	Narrative / mechanistic review	Myocardial interstitial fibrosis in heart failure
8.	Yamada et al.	2025	10.1161/CIRCULATIONAHA.123.067504	Experimental – animal model	Cardiac reprogramming and Gata4 overexpression reduce fibrosis in HFpEF
9.	Kazbanov et al.	2016	10.1038/srep20835	Experimental – computational model	Effects of heterogeneous diffuse fibrosis on arrhythmia dynamics
10.	Travers et al.	2022	10.1172/JCI148554	Narrative / mechanistic review	Therapeutic targets for cardiac fibrosis
11.	Zhang et al.	2024	10.1016/j.biopha.2024.116413	Narrative / mechanistic review	MAPK-ERK/JNK pathway as intervention mechanism of myocardial fibrosis
12.	Tan LG et al.	2015	10.1186/s12872-015-0103-4	Experimental – cell / animal model	PEP-1-SOD1 fusion proteins block myofibroblast activation
13.	Tan R et al.	2023	10.1016/j.phymed.2022.154590	Experimental – animal model	Sodium houttuyfonate against cardiac fibrosis in isoproterenol-induced HF
14.	López et al.	2021	10.1038/s41569-020-00504-1	Narrative / mechanistic review	Diffuse myocardial fibrosis: mechanisms, diagnosis and therapeutic approaches
15.	Lunde et al.	2024	10.1016/j.matbio.2024.08.008	Narrative / mechanistic review	Myocardial fibrosis from the perspective of the ECM: mechanisms to clinical impact
16.	Raafs et al.	2023	10.3390/jcm12175695	Clinical observational study	Collagen biomarkers, LV function and prognosis in DCM (multi-modal study)

17.	Trippel et al.	2018	10.1002/ejhf.960	Clinical interventional study / RCT	PICP-targeted collagen turnover in diabetic HFpEF (DROP-PIP Trial)
18.	Yang et al.	2019	10.1016/j.carpath.2019.107150	Clinical observational study	PICP and MMP-2 as biomarkers of myocardial fibrosis in HCM
19.	López et al.	2016	10.1016/j.jacc.2015.10.063	Clinical observational study	Myocardial collagen cross-linking and HF hospitalization in hypertensive HF
20.	Ferreira et al.	2024	10.1002/ejhf.3101	Clinical interventional study / RCT post-hoc	Empagliflozin effects on collagen biomarkers (EMPEROR trials)
21.	López et al.	2015	10.1016/j.jacc.2015.04.026	Narrative / mechanistic review	Circulating biomarkers of myocardial fibrosis – translational review
22.	Webber et al.	2020	10.1007/s40119-020-00199-y	Narrative / mechanistic review	Myocardial fibrosis in HF: anti-fibrotic therapies and role of CMR in drug trials
23.	Zannad	2014	10.1161/CIRCHEARTFAILURE.114.001156	Editorial	What is measured by cardiac fibrosis biomarkers and imaging – editorial review
24.	Maruyama & Imanaka-Yoshida	2022	10.3390/ijms23052617	Narrative / mechanistic review	The pathogenesis of cardiac fibrosis: a review of recent progress
25.	Lafuse et al.	2020	10.3390/cells10010051	Narrative / mechanistic review	Role of cardiac macrophages on cardiac inflammation, fibrosis and tissue repair
26.	Ridwan et al.	2023	10.1186/s43044-023-00376-z	Narrative / mechanistic review	Molecular mechanisms underlying cardiac fibrosis in diabetes mellitus
27.	Piek et al.	2019	10.1002/ejhf.1421	Editorial	The vicious cycle of arrhythmia and myocardial fibrosis
28.	Austin et al.	2019	10.1038/s41569-019-0200-7	Narrative / mechanistic review	Molecular mechanisms of arrhythmogenic cardiomyopathy
29.	Schelbert	2019	10.1016/j.hfc.2018.12.009	Narrative / mechanistic review	Myocardial scar and fibrosis – clinical review
30.	Ravassa et al.	2023	10.1016/j.mam.2023.101194	Narrative / mechanistic review	Cardiac fibrosis in HF: non-invasive diagnosis and emerging therapeutic strategies
31.	Talman & Ruskoaho	2016	10.1007/s00441-016-2431-9	Narrative / mechanistic review	Cardiac fibrosis in MI: from repair and remodeling to regeneration
32.	Prabhu & Frangogiannis	2016	10.1161/CIRCRESAHA.116.303577	Narrative / mechanistic review	The biological basis for cardiac repair after myocardial infarction
33.	Venugopal et al.	2022	10.3390/cells11091386	Narrative / mechanistic review	Properties and functions of fibroblasts and myofibroblasts in MI
34.	Francis Stuart et al.	2016	10.1016/j.yjmcc.2015.12.024	Narrative / mechanistic review	Crossroads of inflammation, fibrosis and arrhythmia following MI

35.	Shirakawa et al.	2018	10.1161/CIRCULATIONAHA.118.035047	Experimental – animal / translational	IL-10–STAT3–Gal-3 axis in osteopontin-producing macrophage polarization after MI
36.	Wang et al.	2023	10.7150/thno.78736	Experimental – animal model	Hypoxia-induced M2 macrophages and cardiac fibrosis after MI
37.	Weng et al.	2023	10.1161/CIRCRESAHA.123.322596	Experimental – animal model	TGF-β1/SMAD3 regulates PDCD5 suppressing cardiac fibrosis post-MI
38.	Ding et al.	2020	10.14336/AD.2020.0604	Narrative / mechanistic review	Roles of biomarkers in myocardial fibrosis
39.	Borgeat et al.	2015	10.1111/jsap.12332	Veterinary clinical observational study	CITP and HCM mutation status in Ragdoll cats (veterinary clinical study)
40.	Ravassa et al.	2022	10.1002/ejhf.2394	Clinical interventional study / RCT substudy	Collagen cross-linking biomarker assessment and spironolactone response (HOMAGE)
41.	Wirtz & von Känel	2017	10.1007/s11886-017-0919-x	Narrative / mechanistic review	Psychological stress, inflammation and coronary heart disease
42.	Severino et al.	2020	10.3390/ijms21093167	Narrative / mechanistic review	Ischemic heart disease, HF and coronary ion channels
43.	Paulus	2023	10.1161/CIRCIMAGING.123.015732	Editorial	Early myocardial fibrosis in restrictive diabetic cardiomyopathy – editorial
44.	Pan et al.	2023	10.3390/ijms24108604	Narrative / mechanistic review	Role of cardiac fibrosis in diabetic cardiomyopathy
45.	Cheng et al.	2023	10.3389/fendo.2023.1162754	Narrative / mechanistic review	Central role of cardiac fibroblasts in myocardial fibrosis of diabetic cardiomyopathy
46.	Dong et al.	2017	10.3892/ijmm.2017.3311	Narrative / mechanistic review	Galectin-3 as a novel biomarker and target for therapy
47.	Nagao et al.	2018	10.1002/ehf2.12360	Clinical observational study	Circulating collagen type I, III and IV markers vs myocardial collagen mRNA in DCM
48.	Huerta et al.	2016	10.1097/HJH.0000000000000757	Clinical observational study	Cystatin C, TIMP-1 and osteopontin in HFpEF in elderly hypertensive patients
49.	López et al.	2015	10.1002/ejhf.246	Clinical observational study	Galectin-3 and histological/biochemical aspects of myocardial fibrosis in hypertensive HF
50.	Robinson et al.	2023	10.1093/infdis/jiad149	Experimental – animal / translational	Osteopontin as mediator of cardiac interstitial fibrosis in HIV/SIV models
51.	Lepojärvi et al.	2015	10.3389/fphys.2015.00200	Clinical observational study	Serum PINP, PIIINP, Gal-3 and ST2 as surrogates of myocardial fibrosis in stable CAD
52.	Greene et al.	2023	10.1016/j.jacc.2022.11.023	Narrative / mechanistic review	Worsening heart failure: nomenclature, epidemiology and future directions

53.	Baman & Ahmad	2020	10.1001/jama.2020.13310	Narrative / mechanistic review	Heart failure – clinical primer
54.	Oppedisano et al.	2021	10.3390/nu13092965	Narrative / mechanistic review	PUFA supplementation and heart failure: effects on fibrosis and cardiac remodeling
55.	Frangogiannis	2021	10.1093/cvr/cvaa324	Narrative / mechanistic review	Cardiac fibrosis – comprehensive mechanistic review
56.	Simmonds et al.	2020	10.3390/cells9010242	Narrative / mechanistic review	Cellular and molecular differences between HFpEF and HFrEF
57.	Smereka & Ezekowitz	2024	10.1139/cjpp-2023-0403	Narrative / mechanistic review	HFpEF and sex: understanding the role of sex differences
58.	Suthahar et al.	2020	10.1002/ejhf.1771	Narrative / mechanistic review	Sex-related differences in contemporary biomarkers for heart failure
59.	Lau et al.	2023	10.1016/j.jacadv.2023.100332	Narrative / mechanistic review	Sex and age differences in myocardial fibrosis
60.	Lampejo et al.	2021	10.7861/clinmed.2021-0121	Narrative / mechanistic review	Acute myocarditis: aetiology, diagnosis and management
61.	Becher et al.	2017	10.1155/2017/6590609	Experimental – animal model	Cardiac function after acute experimental viral myocarditis
62.	Wang et al.	2018	10.1016/j.bbrc.2018.09.092	Experimental – animal model	Androgen receptor and cardiac fibrosis in experimental autoimmune myocarditis
63.	Tkacz et al.	2020	10.3390/cells9030700	Experimental – animal model	ROCK1+/- and ROCK2+/- mice in experimental autoimmune myocarditis
64.	Tan et al.	2023	10.1007/s10753-023-01809-2	Experimental – animal model	sST2 bridging senescence and TGF-β1/Smad2/3 in cardiac fibrosis in viral myocarditis
65.	Seropian et al.	2024	10.1016/j.cytogfr.2024.10.002	Narrative / mechanistic review	Central role of galectin-3 at crossroads of cardiac inflammation and fibrosis
66.	Screever et al.	2023	10.3390/biom13030410	Clinical observational study	Diffuse myocardial fibrosis on CMR related to galectin-3 and outcome in HF
67.	Pichler et al.	2020	10.1097/HJH.0000000000002504	Clinical observational study	CMR-derived fibrosis, strain and molecular biomarkers in hypertensive heart disease
68.	Prasad & Halliday	2021	10.1016/j.jcmg.2021.03.015	Editorial	Myocardial fibrosis in dilated cardiomyopathy – editorial review
69.	Liu et al.	2022	10.1148/radiol.2021210914	Clinical observational study	Patterns of replacement fibrosis in hypertrophic cardiomyopathy (imaging study)
70.	Sara	2019	10.5935/abc.20190043	Editorial	Myocardial fibrosis in HCM: what remains to be proven – editorial

71.	Bengel & Ross	2019	10.1007/s12350-018-1356-y	Narrative / mechanistic review	Emerging imaging targets for infiltrative cardiomyopathy
72.	Corrado et al.	2024	10.1016/j.ijcard.2023.131447	Clinical guideline / consensus statement	Proposed diagnostic criteria for arrhythmogenic cardiomyopathy (ESC Task Force)
73.	Nguyen et al.	2017	10.1002/cphy.c160046	Narrative / mechanistic review	Cardiac fibrosis and arrhythmogenesis
74.	Disertori et al.	2017	10.1016/j.tcm.2017.01.011	Narrative / mechanistic review	Myocardial fibrosis predicts ventricular tachyarrhythmias
75.	Nagata et al.	2023	10.1161/CIRCIMAGING.122.014963	Clinical observational study	Abnormal mechanics, myocardial fibrosis and ventricular arrhythmias in MVP
76.	Nattel	2017	10.1016/j.jacep.2017.03.002	Narrative / mechanistic review	Molecular and cellular mechanisms of atrial fibrosis in atrial fibrillation
77.	Marrouche et al.	2014	10.1001/jama.2014.3	Clinical observational study	DECAAF: atrial fibrosis by DE-CMR and AF catheter ablation outcomes
78.	Marrouche et al.	2022	10.1001/jama.2022.8831	Clinical interventional study / RCT	DECAAF II: MRI-guided fibrosis ablation vs conventional catheter ablation in AF
79.	Al-Aidarous et al.	2024	10.1136/heartjnl-2023-322612	Narrative / mechanistic review	Management of arrhythmogenic right ventricular cardiomyopathy
80.	Tridimas et al.	2021	10.1177/00045632211025567	Clinical methodological / assay-comparison study	PINP assay comparison in chronic kidney disease (methodological clinical study)
81.	Seo et al.	2017	10.1038/s41598-017-16290-9	Experimental – methodological	Recombinant PICP production and sandwich ELISA development
82.	Kobayashi et al.	2022	10.1002/ejhf.2579	Clinical interventional study / RCT substudy	Type I collagen synthesis markers and echocardiographic response to spironolactone (HOMAGE)
83.	Ferreira et al.	2019	10.1136/heartjnl-2018-313182	Clinical interventional study / RCT substudy	Spironolactone effects on collagen biomarkers in uncontrolled BP (ASCOT substudy)
84.	Safdar et al.	2014	10.1016/j.jchf.2014.03.013	Clinical observational study	Circulating collagen biomarkers in pulmonary arterial hypertension
85.	Lebedev et al.	2020	10.1155/2020/6976153	Clinical observational study	T2DM and HFmrEF/HFpEF: fibrosis biomarkers including PICP/PIIINP ratio
86.	Ionin et al.	2020	10.3390/ijms21165689	Clinical observational study	Galectin-3, PINP and PIIINP in atrial fibrillation and metabolic syndrome
87.	Kawasaki et al.	2017	10.1111/pace.13169	Clinical interventional study / RCT	Eplerenone and atrial fibrosis biomarkers (PINP/PIIINP) in hypertension

88.	Kallergis et al.	2014	10.1093/europace/eut401	Clinical observational study	Sinus rhythm restoration and collagen turnover (CITP) in persistent AF
89.	Fukui et al.	2016	10.1007/s00380-014-0597-1	Clinical observational study	Collagen metabolism (CITP) and LV recovery after beta-blocker therapy in CHF
90.	Wang & Khalil	2018	10.1016/bs.apha.2017.08.002	Narrative / mechanistic review	Matrix metalloproteinases, vascular remodeling and vascular disease
91.	Giannandrea & Parks	2014	10.1242/dmm.012062	Narrative / mechanistic review	Diverse functions of matrix metalloproteinases during fibrosis
92.	Münch et al.	2016	10.1016/j.cardfail.2016.03.010	Clinical observational study	Serum MMPs as biomarkers of myocardial fibrosis and SCD risk in HCM
93.	Lin et al.	2017	10.18632/oncotarget.13795	Clinical observational study	Serum fibrosis markers and restrictive ventricular filling in HFrEF
94.	Kostov & Blazhev	2022	10.3390/bioengineering9030119	Clinical observational study	MMP-1 and TIMP-1 in patients with essential hypertension
95.	Romero et al.	2021	10.1111/jch.14206	Clinical interventional study / RCT post-hoc	ACE inhibitors increase anti-fibrotic biomarkers in LVH
96.	Nagase & Brew	2003	10.1042/bss0700201	Experimental – methodological	Designing TIMP variants as selective metalloproteinase inhibitors
97.	Coates-Park et al.	2024	10.1152/ajpcell.00699.2023	Narrative / mechanistic review	The TIMP protein family: diverse roles in pathophysiology
98.	Yu et al.	2017	N/A – no DOI available	Clinical observational study	MMP-1, TIMP-1 and TGF-β1 in valve tissue of rheumatic heart disease
99.	Vanhoutte & Heymans	2010	10.1016/j.yjmcc.2009.09.013	Narrative / mechanistic review	TIMPs and cardiac remodeling: MMP-independent functions
100.	Seropian et al.	2023	10.3389/fphys.2023.1304735	Narrative / mechanistic review	Unraveling the role of galectin-3 in cardiac pathology and physiology
101.	Berezin & Berezin	2020	10.1155/2020/1215802	Narrative / mechanistic review	Adverse cardiac remodeling after MI: old and new biomarkers
102.	Wang et al.	2023	10.1093/cvr/cvad116	Experimental – animal / cell model	Galectin-3 inhibition post-infarction and progressive cardiac fibrosis
103.	Martínez-Martínez et al.	2019	10.1161/HYPERTENSIONAHA.118.11874	Experimental – animal / translational	CT-1–Gal-3 axis in cardiac fibrosis and inflammation
104.	Lebedev et al.	2021	10.1155/2021/9589185	Clinical observational study	Fibrosis markers (Gal-3) and HF incidence in type 2 diabetes mellitus

105.	Sherpa et al.	2023	10.3390/cells12091218	Clinical autopsy / translational observational study	Galectin-3, cardiac fibrosis and increased risk of sudden death
106.	U.S. FDA	2010	N/A – regulatory document	Regulatory document	BGM Galectin-3 Assay (K093758) – FDA 510(k) clearence
107.	Abdelaziz Mohamed et al.	2019	10.3390/cells8121558	Narrative / mechanistic review	Osteopontin: a promising therapeutic target in cardiac fibrosis
108.	Sawaki et al.	2018	10.1161/CIRCULATIONAHA.117.031358	Experimental – animal model	Visceral adipose tissue drives cardiac aging via osteopontin and fibroblast senescence
109.	Hoeft et al.	2023	10.1016/j.celrep.2023.112131	Experimental – translational	Platelet-instructed SPP1+ macrophages drive myofibroblast activation in fibrosis
110.	Fu et al.	2023	10.1161/ATVBAHA.123.319828	Clinical / translational human tissue study	scRNA-seq: SPP1+ macrophage subpopulation in end-stage heart failure patients
111.	Freiholtz et al.	2023	10.1007/s00109-023-02370-z	Clinical / translational observational study	SPP1/osteopontin as driver of fibrosis in degenerative ascending aortic aneurysm
112.	Lozhkin et al.	2022	10.1016/j.redox.2022.102474	Experimental – animal model	Mitochondrial oxidative stress and diastolic dysfunction via impaired mitochondrial dynamics
113.	Vianello et al.	2019	10.1016/j.biocel.2019.105619	Narrative / mechanistic review	ST2/IL-33 signaling in cardiac fibrosis
114.	Aimo et al.	2019	10.1016/j.jacc.2019.08.1039	Narrative / mechanistic review	Clinical and prognostic significance of sST2 in heart failure
115.	U.S. FDA	2011	N/A – regulatory document	Regulatory document	Presage ST2 Assay (K111452) – FDA 510(k) clearence
116.	McDonagh et al.	2021	10.1093/eurheartj/ehab368	Clinical guideline / consensus statement	2021 ESC Guidelines for diagnosis and treatment of acute and chronic heart failure
117.	Heidenreich et al.	2022	10.1161/CIR.0000000000001063	Clinical guideline / consensus statement	2022 AHA/ACC/HFSA Guideline for the management of heart failure
118.	Liu et al.	2017	10.1016/j.jacc.2017.10.044	Clinical observational study	NT-proBNP and myocardial fibrosis in MESA population study
119.	Zhao et al.	2014	10.1016/j.jjcc.2013.09.013	Clinical observational study	Periostin expression and myocardial fibrosis in human failing hearts
120.	Shimazaki et al.	2008	10.1084/jem.20071297	Experimental – animal model	Periostin is essential for cardiac healing after acute MI

121.	Kruger et al.	2014	10.1371/journal.pone.0101522	Clinical observational study	Fibulin-1, NT-proBNP and suPAR in aortic valve stenosis (SEAS study)
122.	Imanaka-Yoshida et al.	2020	10.1152/ajpcell.00353.2020	Narrative / mechanistic review	Tenascin-C in cardiac disease: inflammation, repair and fibrosis
123.	Thum et al.	2008	10.1038/nature07511	Experimental – animal / cell model	MicroRNA-21 in myocardial disease via MAP kinase signaling in fibroblasts
124.	van Rooij et al.	2008	10.1073/pnas.0805038105	Experimental – animal model	miR-29 dysregulation after MI and cardiac fibrosis
125.	Vegter et al.	2016	10.1002/ejhf.495	Narrative / mechanistic review	MicroRNAs in heart failure: from biomarker to target for therapy
126.	Stawski et al.	2014	10.1371/journal.pone.0109763	Experimental – animal model	MMP-12 deficiency attenuates angiotensin II–induced vascular injury and heart fibrosis
127.	Mohamad et al.	2024	10.1016/j.intimp.2024.111963	Experimental – animal model	Sacubitril/valsartan alleviates sunitinib-induced cardiac fibrosis
128.	Virgen-Ortiz et al.	2019	10.3390/medicina55050199	Experimental – animal model	MMP system and fibrosis in rat heart during late pregnancy and postpartum
129.	Blanda et al.	2020	10.3390/ijms21239232	Narrative / mechanistic review	Galectin-3 in cardiovascular diseases
130.	Meijers et al.	2016	10.1016/j.ajpath.2016.05.002	Commentary	Galectin-3, cardiac function and fibrosis – editorial review
131.	Souza et al.	2017	10.1016/j.ajpath.2017.01.016	Experimental – animal / translational	Galectin-3 expression, myocarditis and fibrosis in Chagas disease cardiomyopathy
132.	Zhong et al.	2022	10.1016/j.mvr.2022.104347	Experimental – animal model	Src inhibition, galectin-3 and cardiac fibrosis in AngII-induced hypertrophy
133.	Pozder et al.	2022	10.1016/j.retram.2021.103321	Experimental – cell / animal model	Pectins inhibit galectin-3–related cardiac fibrosis
134.	Dahl et al.	2012	10.1161/JAHA.112.003889	Clinical observational study	Fibulin-1 and restrictive LV filling and mortality in aortic valve stenosis
135.	Ledwoń et al.	2021	10.3390/ma14123217	Narrative / mechanistic review	Peptides and peptidomimetics as inhibitors of fibrillar collagen degradation enzymes
136.	Lewis et al.	2021	10.1038/s41591-021-01452-0	Clinical interventional study / RCT	Pirfenidone in HFpEF: a randomized phase 2 trial
137.	Vistnes	2024	10.3390/ph17030267	Narrative / mechanistic review	Challenges and opportunities for TGF-β inhibition for cardiac fibrosis
138.	Le et al.	2024	10.31083/j.rcm2512447	Narrative / mechanistic review	AI for left ventricle analysis in cardiac magnetic resonance – clinical review

Study types were assigned according to the predominant design and role of each cited source within this targeted narrative review. DOI, digital object identifier; AF, atrial fibrillation; AngII, angiotensin II; ASCOT, Anglo-Scandinavian Cardiac Outcomes Trial; ATF3, activating transcription factor 3; CAD, coronary artery disease; CHF, chronic heart failure; CITP, collagen type I C-terminal telopeptide; CKD, chronic kidney disease; CMR, cardiac magnetic resonance; CT-1, cardiotrophin-1; DCM, dilated cardiomyopathy; DE-CMR, delayed-enhancement cardiac magnetic resonance; DECAAF, Delayed-Enhancement MRI Determinant of Successful Radiofrequency Catheter Ablation of Atrial Fibrillation; ECM, extracellular matrix; FDA, U.S. Food and Drug Administration; Gal-3, galectin-3; HCM, hypertrophic cardiomyopathy; HF, heart failure; HFmrEF, heart failure with mildly reduced ejection fraction; HFpEF, heart failure with preserved ejection fraction; HFrEF, heart failure with reduced ejection fraction; HIV, human immunodeficiency virus; HOMAGE, Heart Omics in AGEing; IL, interleukin; JNK, c-Jun N-terminal kinase; LV, left ventricular; MAPK-ERK, mitogen-activated protein kinase–extracellular signal-regulated kinase; MESA, Multi-Ethnic Study of Atherosclerosis; MI, myocardial infarction; MMP, matrix metalloproteinase; MMP-2, matrix metalloproteinase-2; MVP, mitral valve prolapse; NT-proBNP, N-terminal pro-B-type natriuretic peptide; PICP, procollagen type I C-terminal propeptide; PINP, procollagen type I N-terminal propeptide; PIIINP, procollagen type III N-terminal propeptide; PTX3, pentraxin 3; RCT, randomized controlled trial; SCD, sudden cardiac death; scRNA-seq, single-cell RNA sequencing; SIV, simian immunodeficiency virus; SMAD, suppressor of mothers against decapentaplegic; SOD1, superoxide dismutase 1; SPP1, secreted phosphoprotein 1; sST2, soluble suppression of tumorigenicity 2; ST2, suppression of tumorigenicity 2; suPAR, soluble urokinase plasminogen activator receptor; T2DM, type 2 diabetes mellitus; TGF-β, transforming growth factor beta; TIMP-1, tissue inhibitor of metalloproteinases-1.

Supplementary Table S3. Reference classification by study type and topic domain (n = 138).

**Study type**	**n**	**%**
Narrative / mechanistic review	56	40.6%
Experimental study (preclinical; animal; computational)	31	22.5%
Clinical observational / translational study	31	22.5%
Clinical interventional / RCT / post-hoc analysis	9	6.5%
Editorial / commentary	6	4.3%
Clinical guideline / consensus statement	3	2.2%
Regulatory document	2	1.4%

Percentages were calculated from the final revised reference list (n = 138). Study types were assigned according to the predominant design and role of each cited source. Clinical observational/translational studies include human cohort, imaging, biomarker, autopsy, and tissue-based studies; experimental studies include animal, computational, and methodological studies. These categories describe the cited evidence base of a targeted narrative review and should not be interpreted as PRISMA-defined included studies.
